# The mouse DXZ4 homolog retains Ctcf binding and proximity to *Pls3 *despite substantial organizational differences compared to the primate macrosatellite

**DOI:** 10.1186/gb-2012-13-8-r70

**Published:** 2012-08-20

**Authors:** Andrea H Horakova, J Mauro Calabrese, Christine R McLaughlin, Deanna C Tremblay, Terry Magnuson, Brian P Chadwick

**Affiliations:** 1 Department of Biological Science, Florida State University, Tallahassee, FL 32306-4295, USA; 2 Department of Genetics, Carolina Center for Genome Sciences and Lineberger Comprehensive Cancer Center, University of North Carolina, Chapel Hill, NC 27599, USA

## Abstract

**Background:**

The X-linked macrosatellite DXZ4 is a large homogenous tandem repeat that in females adopts an alternative chromatin organization on the primate X chromosome in response to X-chromosome inactivation. It is packaged into heterochromatin on the active X chromosome but into euchromatin and bound by the epigenetic organizer protein CTCF on the inactive X chromosome. Because its DNA sequence diverges rapidly beyond the New World monkeys, the existence of DXZ4 outside the primate lineage is unknown.

**Results:**

Here we extend our comparative genome analysis and report the identification and characterization of the mouse homolog of the macrosatellite. Furthermore, we provide evidence of DXZ4 in a conserved location downstream of the *PLS3 *gene in a diverse group of mammals, and reveal that DNA sequence conservation is restricted to the CTCF binding motif, supporting a central role for this protein at this locus. However, many features that characterize primate DXZ4 differ in mouse, including the overall size of the array, the mode of transcription, the chromatin organization and conservation between adjacent repeat units of DNA sequence and length. Ctcf binds Dxz4 but is not exclusive to the inactive X chromosome, as evidenced by association in some males and equal binding to both X chromosomes in trophoblast stem cells.

**Conclusions:**

Characterization of Dxz4 reveals substantial differences in the organization of DNA sequence, chromatin packaging, and the mode of transcription, so the potential roles performed by this sequence in mouse have probably diverged from those on the primate X chromosome.

## Background

Over two-thirds of the human genome is likely to be composed of repetitive DNA [1], of which a significant proportion is tandem repeat DNA [2]. The tandem repeats consist of homologous DNA sequences arranged head to tail, and the number of repeat units is invariably polymorphic from one individual to the next [3]. The size of the individual repeat unit varies substantially, from the simple microsatellite composed of individual repeat units of 1 to 6 bp spanning tens to hundreds of base pairs [4] to those consisting of individual repeat units of several kilobases that can cover hundreds to thousands of kilobases [5]. For some tandem repeat DNA, deciphering of function is assisted by location, such as the alpha satellite DNA that defines active centromeres [6] to the telomeric minisatellite [7], but the roles of others in our genome remain unknown, resulting in opinions in the past that they serve no purpose [8,9].

Despite a lack of functional understanding for these sequences, their contribution to disease susceptibility is obvious, as is demonstrated by the devastating impact of simple repeat expansions [10] or macrosatellite contraction [11,12].

Macrosatellites are tandem repeat DNA with some of the largest individual repeat units (most >2 kb), which can extend over hundreds to thousands of kilobases [5,11,13-17]. Most occupy specific locations on one or two chromosomes, like the X-linked macrosatellite DXZ4, which is unique to Xq23 [14]. Because of its physical location on the X chromosome, DXZ4 is exposed to the process of X-chromosome inactivation (XCI). XCI is the mammalian form of dosage compensation, an epigenetic process that serves to balance the levels of X-linked gene expression in the two sexes [18]. It occurs early in female development and shuts down gene expression from the X chromosome (Xi) chosen to become inactive by repackaging the DNA into facultative heterochromatin [19]. One characteristic difference between Xi chromatin and that of the active X chromosome (Xa) is hypermethylation of cytosine residues at CpG islands (CGIs) [20,21], but DXZ4, which is itself one of the largest CGIs in the human genome, does not conform. Instead, DXZ4 CpG residues are hypomethylated on the Xi and hypermethylated on the Xa [14,22]. Consistent with the DNA methylation profile of DXZ4, its nucleosomes are characterized by the heterochromatin-associated modification histone H3 trimethylated at lysine 9 [23,24] on the Xa and the euchromatin-associated modification histone H3 dimethylated at lysine 4 (H3K4me2) [23] on the Xi [22,25]. Furthermore, the multifunctional zinc-finger protein CCCTC-binding factor (CTCF) [26] associates specifically with the euchromatic form of DXZ4 on the Xi [22,27]. The role DXZ4 performs on the Xi when packaged as CTCF-bound euchromatin flanked by heterochromatin or on the Xa and male X chromosome when packaged into heterochromatin flanked by euchromatin remains unclear. However, we have recently shown that, in humans, DXZ4 mediates Xi-specific CTCF-dependent long-range intrachromosomal interactions with other tandem repeat DNA [28], suggesting a structural role for DXZ4 that may orchestrate the alternative three-dimensional organization of the Xi relative to the Xa [29]. To gain insight into DXZ4 function, we previously investigated DXZ4 in a variety of representative primates and found that CTCF binding at the Xi was conserved, as were the chromatin organization, expression, and arrangement of the macrosatellite into large homogenous tandem arrays [30], but beyond the New World monkey branch, primary DNA sequence composition and tandem-repeat unit size diverged rapidly from that observed in humans, with the notable exception of a relatively small proportion of DXZ4 that encompassed the CTCF binding site and promoter element [22,30]. To further our understanding of DXZ4, we extended our analysis beyond the primate lineage in an attempt to identify a homolog of DXZ4 in mouse. Mouse has been the logical model organism of choice for investigation of XCI, and much of what we understand about the process has been obtained through mouse manipulations *in vivo *and *in vitro *[31]. Despite differences in the early stages of XCI between humans and mice [32], and differences in the extent of escape from XCI [33,34], identification of a mouse homolog of DXZ4 would provide a tractable system in which to investigate function. Here we report the identification and characterization of the mouse homolog of DXZ4. We show that DNA sequence conservation is restricted to a short DNA sequence corresponding to the CTCF binding site, but many features of DXZ4 differ substantially in the mouse, and as a result manipulation of mouse Dxz4 is unlikely to provide insight into all aspects of DXZ4 function in primates.

## Results and discussion

### Genomic organization of a mouse candidate for Dxz4

**Figure 1:**
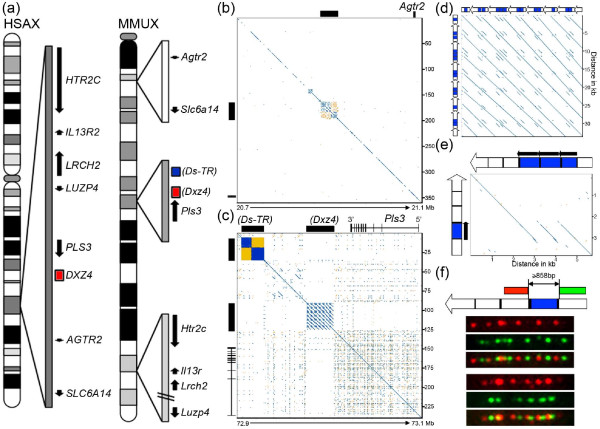
**Genomic characterization of the mouse Dxz4 locus**. **(a) **Ideograms of the human (HSAX) and mouse (MMUX) X chromosomes. Regions relevant to the search for Dxz4 are expanded to the right of the chromosome. Genes are represented by solid arrows pointing in the direction of transcription. Length represents extent of the gene. Human DXZ4 is represented as the red box. The location of the putative Dxz4 homolog and the downstream tandem repeat are highlighted proximal to mouse *Pls3 *as red and blue boxes, respectively. **(b) **Pair-wise alignment of approximately 360 kb (scale in kilobases given on the y-axis) downstream of the mouse *Agtr2 *gene (20.7 to 21.1 Mb, mm9, indicated for the x-axis). Sequence similarity is shown in blue with inverted similarity in yellow. Black bars on the top and left edges indicate extensive repeats. **(c) **Pair-wise alignment of approximately 240 kb encompassing the *Pls3 *gene (72.9 to 73.1 Mb, mm9) and distal sequence. **(d) **Pairwise alignment of the 36-kb mouse Dxz4 array. The block arrows on the top and left edges represent Dxz4 tandem repeat monomers. **(e) **Pairwise alignment of the largest and smallest Dxz4 monomers (block arrows on top and left edges) highlighting the existence of an internal variable number tandem repeat (VNTR) represented by the black arrows above the blue boxes. Perpendicular black lines within the monomers indicate the locations of simple repeats. **(f) **Extended DNA fiber fluorescence *in situ *hybridization (FISH) of the Dxz4 array. At the top is a schematic of a single Dxz4 monomer. The regions of Dxz4 used to generate direct-labeled FISH probes are indicated to the left (red) and right (green) of the VNTR (blue). Immediately below are examples of hybridization results. All pairwise alignments used the DNA sequence compared with a repeat-masked version of itself with the exception of that shown in (c), which compared non-repeat-masked sequences to show the inverted satellite repeat. Alignments were all made with YASS [71], and the output was pseudocolored to avoid red-green.

A comparison of a human DXZ4 3.0-kb tandem repeat monomer against the assembled mouse genome (mm9) with Blast-Like Alignment Tool (BLAT) produced no significant matches on the Ensembl genome browser [35] and a limited number of autosomal and X-linked matches on the UCSC Genome Browser [36] (data not shown). We therefore explored conserved gene order in human and mouse to identify a DXZ4 homolog [37]. DXZ4 resides at Xq23 [14] and is located between the t-Plastin gene (*PLS3*) and the Angiotensin II receptor, type-2 gene (*AGTR2*) (Figure 1a). Comparative analysis of human genes in the vicinity of DXZ4 in the mouse genome revealed several differences in the gene order (Figure 1a), including a break point between the mouse *PLS3 *and *AGTR2 *orthologs *Pls3 *and *Agtr2 *and between *Pls3 *and the mouse ortholog of *HTR2C*. In mouse, the nearest proximal gene to *Pls3 *is Rab39b (>200 kb proximal), whereas the nearest distal gene is *Tbl1x*, located 1.8 Mb distal to *Pls3*. In humans, the respective orthologs of these two genes are located >39 Mb distal to and >20 Mb proximal to *PLS3*, indicating that *Pls3 *alone defines the block of synteny for this region with the human X chromosome. In primates, DXZ4 is a homogenous tandem repeat [17,30]; we therefore performed pair-wise alignments of the genomic DNA sequence upstream of *Agtr2 *and downstream of *Pls3 *to look for evidence of tandem repeat DNA. Approximately 150 kb upstream of *Agtr2*, we identified an inverted repeat (Figure 1b) but no obvious tandem repeats. In contrast, pairwise alignments of the genomic DNA sequence distal to *Pls3 *identified an extensive tandem repeat spanning approximately 35 kb located 19 kb 3' to *Pls3 *(Figure 1c). In addition, an extensive minisatellite sequence spanning approximately 30 kb was located a further 24 kb downstream of the tandem repeat. The minisatellite was composed primarily of a novel gamma satellite sequence that is interrupted by several L1 and SYNREP repetitive elements, and the sequence itself displayed an inversion almost midway through the locus (Additional file 1a). The repeat showed significant sequence matches only to the mouse X chromosome, and no homologous repeat exists on the human or rat X chromosomes (data not shown). We therefore focused primarily on the tandem repeat.

We next checked to see how frequently a tandem repeat of comparable size (35 kb) occurred on the mouse X chromosome to see if detection of such a sequence downstream of *Pls3 *would likely occur by chance. Pair-wise alignments along the length of the mouse X chromosome indicated that large tandem repeats are not common (Additional file 2), supporting the possibility that this might be the mouse homolog of DXZ4.

Pair-wise alignment of the tandem repeat sequence revealed that, unlike DXZ4 in primates, where repeat units are very similar in size within a species [17,30], the individual repeating units of the mouse tandem repeat varied from 3.8 to 5.7 kb (Figure 1d). Closer examination showed that the size variation was accounted for by the presence of an internal variable number tandem repeat (VNTR) of an approximately 900-bp sequence present as between one and three copies per monomer (Figure 1e). As in primate DXZ4 [14,17,30], less than 6% of the smallest monomer DNA sequence (3.8 kb) was repeat masked, and all of the masked regions corresponded to simple repeats. Examination of the largest monomer (5.7 kb) revealed that the first 147 bp of the internal VNTR was derived from an ERV class II long terminal repeat and that the other edge of the VNTR is defined by a simple repeat. The location of these repeat sequences may contribute to the observed copy-number variation. Three other defining features of human DXZ4 were examined for the novel mouse tandem repeat: CpG content, sequence variation between monomers, and size of the tandem array. Human DXZ4 DNA is 62.2% GC, contains 186 CpG dinucleotides per monomer [38], and shows less than 1% sequence divergence between adjacent monomers [17]. In contrast, the mouse 3.8-kb monomer is 53.4% GC, contains 36 CpG dinucleotides, and shows greater than 5% sequence divergence from other monomers in the tandem array. In primates, DXZ4 is composed of as many as 100 repeat units spanning hundreds of kilobases on the X chromosome [14,17]. In the current build of the mouse genome, the tandem repeat is composed of approximately seven repeat units. Given the inherent difficulty with the computer-based assembly of tandem repeats [39], the actual array could be more extensive. We have previously used extended DNA fiber fluorescence *in situ *hybridization (FISH) to confirm tandem arrangement and copy-number variation of human DXZ4 [17]. We applied the same procedure to examine such variation in the mouse tandem repeat, revealing approximately six tandem repeats in two independent mouse cell lines (Figure 1f). This result suggested that the mouse tandem repeat is relatively small, and the presence of the tandem repeat and extensive flanking DNA sequences entirely within the inserts of at least ten independent mouse bacterial artificial chromosomes from three different libraries derived from two *Mus musculus *subspecies lends additional support (Additional file 3). The logical interpretation of these observations was that the mouse sequence downstream of *Pls3 *is a tandem repeat but that the overall copy number of repeat units is low, resulting in a smaller array than in primates. Despite these differences from primate DXZ4, the tandem repeat remains a good candidate for the mouse homolog and from this point forward is referred to as Dxz4.

### Expression of Dxz4

**Figure 2:**
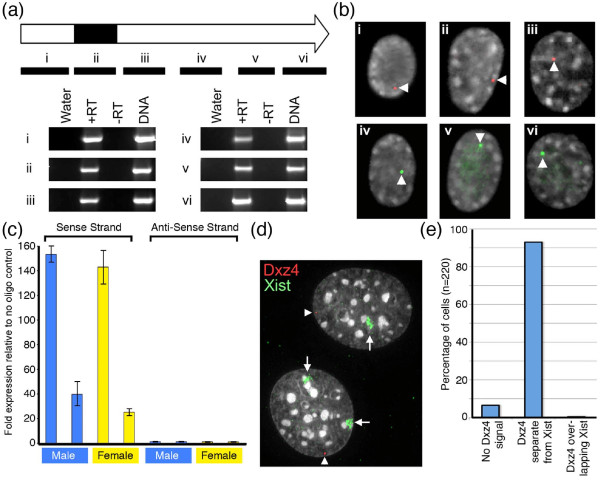
**Characterization of unspliced Dxz4 transcript**. **(a) **Schematic map of a Dxz4 monomer. The internal VNTR is represented by the black box. Below it are indicated six intervals (i to vi) assessed by reverse-transcription PCR (RT-PCR). The RT-PCR results for i to vi are given as images of ethidium bromide-stained agarose gels for NIH/3T3 complementary DNA (cDNA). Samples include water (W), RNA incubated with (+RT) and without (-RT) reverse transcriptase, and genomic DNA. **(b) **RNA FISH results of direct-labeled Spectrum-Orange or Spectrum-Green probes for regions i to vi in NIH/3T3 cells. Signals are indicated by white arrows merged with DAPI (black and white). **(c) **Strand-specific quantitative RT-PCR analysis of Dxz4 expression in two independent male and female samples. Graph shows fold expression of dxz4 in sense (left) and anti-sense (right) primed cDNA relative to cDNA prepared with no gene-specific primer. Error bars show standard deviation. **(d) **RNA FISH analysis of unspliced Dxz4 (red) and Xist RNA (green) merged with DAPI (black and white) in female cells. Dxz4 indicated by the white arrowheads and inactive X chromosome-specific transcript (Xist) by the white arrows. **(e) **Frequency of Dxz4 RNA FISH signals overlapping Xist in female cells.

Primate DXZ4 is expressed, and all regions of a monomer can be detected in complementary DNA (cDNA) [17,22,30]. Six regions of Dxz4 were assessed in cDNA from several different mouse total-RNA sources. The example shown in Figure 2a indicates that mouse Dxz4 was also expressed and that all parts of the Dxz4 monomer are transcribed into RNA. This result was confirmed by RNA FISH showing readily detectable Dxz4 primary transcript by means of direct-labeled Dxz4 probes (Figure 2b). In humans, DXZ4 is primarily transcribed from one strand (since designated the sense strand), but antisense transcript can be detected in females and is therefore interpreted as originating from the Xi [22]. Our previous data showed that only sense transcript could be detected in macaque [30]. To assess the relative frequencies of sense and antisense transcription of Dxz4, we primed male and female cDNA from total RNA using oligonucleotides that would prime sense or antisense cDNA synthesis. As in macaque, only sense transcript was readily detected (Figure 2c). In humans, DXZ4 transcript can be detected from the Xa and the Xi [17,22], although expression in macaque is almost exclusively restricted to the Xa [30]. RNA FISH was performed on female mouse cells with a direct-labeled Dxz4 probe and a probe to the X inactive specific transcript (Xist) [40,41] to define the location of the Xi (Figure 2d). As in macaque, Dxz4 could only be readily detected from the Xa (Figure 2e). Collectively, our interpretation of these data is that expression of Dxz4 is restricted to the Xa allele and from one strand only.

**Figure 3:**
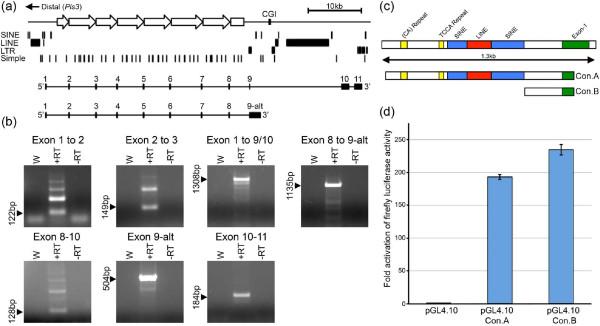
**Expression of spliced Dxz4 and promoter characterization**. **(a) **Schematic map of the Dxz4 region representing 72.95 to 73.01 Mb of the mouse X chromosome (mm9). The map is inverted for simplicity and the distal direction toward *Pls3 *indicated. Open block arrows represent Dxz4 monomers. A downstream CGI is indicated. Immediately below is a map indicating location and type of repeat elements for the interval: LINE, long interspersed nuclear element; LTR, long terminal repeat; SINE, short interspersed nuclear element. Below that are the maps of two putative alternatively spliced transcripts based on expressed sequence tag evidence. **(b) **Confirmation of spliced transcripts by RT-PCR. Each of the seven panels is an image of an ethidium bromide-stained agarose gel showing RT-PCR results for PCR between the exons indicated above. To the left of each image is the predicted product size. Samples include water control (W) and RNA incubated with (+RT) and without (-RT) reverse transcriptase. **(c) **DNA sequence feature map of the 1.3-kb region immediately upstream of Dxz4 exon 1 (green). Repetitive elements are indicated above the corresponding colored boxes. Immediately below are the regions cloned upstream of a promoterless luciferase reporter gene: construct A (Con.A) and construct B (Con.B). **(d) **Luciferase activity measured in NIH/3T3 cell extracts 72 hours after transfection with the promoterless luciferase vector (pGL4.10) or the same vector containing inserts for construct A or B. Fold activation of luciferase is shown to the left. Data represent the mean and standard deviation of replicate experiments each performed in triplicate.

Examination of the GenBank mouse mRNA annotation for the Dxz4 locus on the UCSC Genome Browser [36] revealed the presence of two alternatively spliced transcripts spanning Dxz4. Both transcripts originate at an exon almost 2.2 kb from the distal edge of the array (Figure 3a). The transcript then spliced to the same 163-nucleotide sequence within each of the monomers before splicing to an exon located 1.1 kb proximal to the array. One of the two spliced transcripts proceeded to be spliced to two additional exons approximately 16.0 kb downstream, whereas the other read through the splice site before terminating after a further 2.0 kb. To confirm the existence of the spliced forms of Dxz4, we performed reverse-transcription PCR (RT-PCR) between different combinations of the exons. The anticipated product was detected for each of the RT-PCR experiments (Figure 3b). Furthermore, the RT-PCR confirmed that the transcript contains multiple copies of the 163-nucleotide exon as can be seen from the laddered effect of progressively larger PCR products (see the PCR of exon 1 to 2 or 2 to 3 as examples). Furthermore, this exon was also alternatively spliced with some transcripts omitting one or more 163-nucleotide exons. This result could be observed as smaller laddered bands when RT-PCR was performed across the entire array (Figure 3b, exon 1 to 9/10).

Both the spliced and unspliced transcripts corresponded to the sense transcript, and therefore probably originated from a common promoter, unlike human DXZ4, which contains a region with promoter activity within each monomer [22]. Examination of histone modification profiles from the Encyclopedia of DNA Elements (ENCODE) [42] revealed a distinct peak of histone H3 trimethylated at lysine 4 (H3K4me3) [43] in the vicinity of exon 1 (data not shown). H3K4me3 is a modification associated with transcriptional start sites [44]. We therefore cloned the DNA sequence 5' of Dxz4 exon 1 immediately upstream of a promoterless luciferase reporter gene. Two constructs were generated. The first consisted of a 1.2-kb sequence that contained several repetitive elements that are located immediately upstream of exon 1 (Figure 3c). The second construct consisted of a 238-bp unique sequence 5' of exon 1. Robust promoter activity was detected for both constructs (Figure 3d); the highest activity consistently originated from the smaller unique sequence construct, confirming the location of the minimal Dxz4 promoter.

We next checked to see if the Dxz4 tandem repeat possessed intrinsic promoter activity like human DXZ4 [22]. Two overlapping fragments encompassing a complete Dxz4 monomer were PCR amplified (Additional file 4a), TA cloned and sequence verified. The DNA was then subcloned upstream of the promoterless luciferase reporter gene and assessed for promoter activity alongside the Dxz4 minimal promoter described above. Neither Dxz4 fragment showed obvious activity compared to the Dxz4 minimal promoter that consistently activated luciferase greater than 200-fold over the empty vector (Additional file 4b). Therefore, our interpretation of this result is that both the spliced and unspliced Dxz4 transcripts likely originate from transcription initiating from the minimal promoter. Consequently, it should be possible to detect by RT-PCR a transcript that spans exon 1 directly to the tandem repeat (Additional file 5a). Despite the relatively large size (approximately 2.5 kb) and proximity to the very 5' end of the message, this transcript can be detected in cDNA (Additional file 5b).

When the H3K4me3 profile of Dxz4 was examined, an additional major peak was noticed immediately distal to the downstream inverted tandem repeat (Ds-TR; data not shown), suggesting promoter activity within this region and the possibility that, like Dxz4, the Ds-TR is expressed. RT-PCR confirmed expression of Ds-TR in both male and female samples (Additional file 1b).

### CpG methylation analysis in and around Dxz4

**Figure 4:**
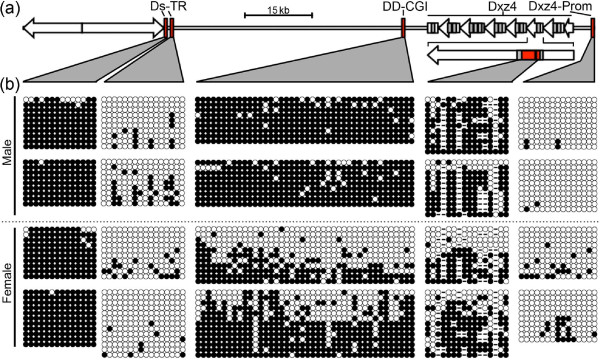
**DNA methylation of elements in the vicinity of Dxz4**. **(a) **Schematic map of the region encompassing Dxz4 and the downstream satellite repeat (diverging open arrows). Left-pointing arrows represent Dxz4, and the location of the Dxz4 promoter and CGI are indicated. The red boxes indicate regions assessed for DNA methylation by PCR of bisulfite-modified DNA, cloning, and sequencing. The location of bisulfite analysis within the Dxz4 array is shown for a single monomer immediately below the array. **(b) **Cytosine methylation at CpG dinucleotides for the five regions shown in (a). Data are given for two independent males (top) and two independent females (bottom). Methylated cytosine is represented by a black circle whereas unmethylated is represented by an open circle. DNA variants that result in a sequence that is no longer a CpG are represented by dashes. Each row of circles represents DNA sequence obtained from a single clone, and each set of data consists of at least nine independent clones.

DXZ4 is unusual in that CpG dinucleotides are hypermethylated on the Xa but hypomethylated on the Xi [14,22], a trait that is conserved in macaque [30]. We selected several regions in and around Dxz4 at which to determine and compare the CpG methylation profiles of males and females (Figure 4a). These sites included the Dxz4 promoter, a region of relatively high CpG incidence within the Dxz4 internal VNTR, a CGI immediately downstream of the Dxz4 array (DD-CGI), and two regions in the vicinity of the H3K4me3 peak adjacent to the Ds-TR.

The Dxz4 promoter (Figure 4b, far right) showed a significantly higher percentage of CpG methylation in females than in males (*P *= 0.0052, two-sample *t*-test). This result is consistent with our expression analysis (Figure 2), suggesting that transcription of Dxz4 is subject to XCI [20,21] and explaining why Dxz4 transcript was only detected from the Xa (Figure 2e).

Males and females did not differ significantly in methylation of the sequence closest to the Ds-TR (profile on far left in Figure 4b; *P *= 0.7580) or in the region immediately distal to it (*P *= 0.0577), but the two regions differed vastly; the proximal sequence was almost entirely methylated, and the distal sequence hypomethylated, on both X chromosomes. Both sites overlap a broad signal of H3K4me3 (data not shown), but examination of other ENCODE features [42] at these two regions revealed that the hypomethylated sequence overlapped a major peak of occupancy for Ctcf [45] and a DNaseI hypersensitive site [46], whereas the hypermethylated site did not (Additional file 6). Binding of Ctcf to target sites containing CpG is sensitive to methylation [47,48]. The hypomethylation in males and females suggests that Ctcf has the potential to bind this region on both the Xa and the Xi.

Males and females did differ significantly in CpG methylation at the Dxz4 array (*P *= 0.0027) similar to what we and others have reported for primate DXZ4 [14,22,30]. However, many sites of CpG residues predicted on the basis of the reference genome sequence (mm9) are not conserved, as demonstrated by the numerous gaps in the bisulfite profiles. Methylated cytosine in CpG is prone to mutation by deamination, whereas mutation rates of unmethylated CpG are lower [49]. As a consequence, hypomethylated CGIs are evolutionarily conserved [50]. The apparent lack of conservation of CpG dinucleotides at Dxz4 is consistent with the overall hypermethylated profiles (Figure 4b). This situation differs from that of primate DXZ4, where CpG residues are highly conserved [22,30], consistent with evolutionary maintenance of DXZ4 as an extensive CGI [50].

Furthermore, males and females did differ significantly in methylation at DD-CGI (*P *= <0.0001); more hypomethylated clones were obtained from the female samples (Figure 4b). Our interpretation of these data is that DD-CGI is hypomethylated on the Xi. DD-CGI spans 333 bp and contains 40 CpGs on the basis of the C57BL/6J reference genome sequence (mm9). None of the genomic feature annotations generated by ENCODE [42], including Ctcf, highlight DD-CGI, and therefore the significance of Xi hypomethylation remains unclear.

### Histone methylation and Ctcf association with sequences in the vicinity of Dxz4

**Figure 5:**
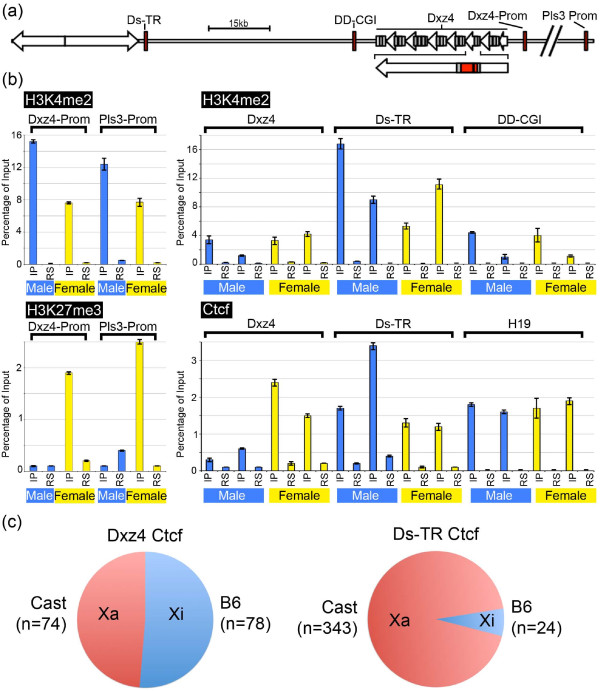
**Characterization of chromatin in the vicinity of Dxz4**. **(a) **Schematic map of the region encompassing Dxz4 and the downstream satellite repeat (diverging open arrows). Left-pointing facing arrows represent Dxz4, and the location of the CGI and promoters for Dxz4 and *Pls3 *are indicated. The angled double strike through the map between the Dxz4 and *Pls3 *promoters represents an approximately 114-kb gap. The red boxes indicate the regions assessed by chromatin immunoprecipitation (ChIP)-PCR. **(b) **Graphs showing results of ChIP assayed by quantitative PCR. The mean and standard deviation for the ChIP elution (IP) and for a negative control rabbit serum (RS) are shown as percentage of the input. For H3K4me2 and H3K27me3 at the Dxz4 promoter (Dxz4-Prom) and *Pls3 *promoter (Pls3-Prom), data for one male and one female are shown. For H3K4me2 and Ctcf at Dxz4, DS-TR and DD-CGI, data are shown for two independent male and female samples. **(c) **Pie charts showing the percentage of C57BL/6J (B6) or castaneous (Cast) informative allele calls for Ctcf ChIP-Seq fragments for Dxz4 and the downstream tandem repeat (Ds-TR) Ctcf binding sites.

Next we sought to complement the DNA methylation analysis by examining histone methylation and Ctcf binding in and around Dxz4. Several sites were selected, including the Dxz4 promoter, the Dxz4 VNTR region, DD-CGI, Ds-TR, and the *Pls3 *promoter as a control for mouse genes subject to XCI [34] (Figure 5a).

Consistent with the expression analysis (Figure 2) and CpG methylation (Figure 4b), the Dxz4 promoter was characterized by the euchromatin mark H3K4me2 in male and female cells, whereas the facultative heterochromatin marker histone H3 trimethylated at lysine 27 (H3K27me3) was only a feature of the female samples (Figure 5b). The same profile is obtained for the *Pls3 *promoter, which is subject to XCI in mouse [34]. Given that genes on the Xi are silenced by H3K27me3 [51,52], these data further support the conclusion that Dxz4 expression is subject to XCI.

In primates, H3K4me2 is a feature of DXZ4 on the Xi [22,30], although this modification can be detected on the male X at low levels in some individuals and as a result of cellular transformation [53]. In contrast, H3K4me2 was readily detected at Dxz4 in males and females (Figure 5b), another difference between mouse and primate DXZ4. Somewhat surprisingly, given the methylation profile at DD-CGI (Figure 4b), H3K4me2 could also be detected at this site in males and females. One possible explanation is that the DD-CGI is located within the transcriptional unit of one of the spliced Dxz4 transcripts (Figure 3a). Therefore, the detection of the euchromatin mark may reflect variable levels of H3K4me2 in the body of active genes [44].

A defining feature of primate DXZ4 is the association of CTCF with the Xi allele [22,30]. Ctcf was readily detected at Dxz4 in multiple independent female samples, but Ctcf was also detected, albeit at lower levels, in some but not all males (Figure 5b and data not shown). To investigate further the relationship between Ctcf and Dxz4 on the Xa and Xi, we examined DNA sequence reads from Ctcf chromatin immunoprecipitation (ChIP) combined with next generation sequencing (ChIP-Seq) performed on trophoblast stem cells (TSCs), which are derived from the extraembryonic material and undergo imprinted XCI with preferential inactivation of the paternal X chromosome [54]. The TSCs were derived from a cross of a male C57BL/6J (BL6) with a female castaneous (cast) mouse. As a result, the BL6 X chromosome will be the Xi. ChIP-Seq reads were compared to BL6 and cast variant sequences for the Dxz4 interval assessed by ChIP-PCR and, where informative, were designated as originating from the Xa (cast) or Xi (BL6). Of 152 ChIP-Seq reads, almost half were assigned to the Xa and half to the Xi (Figure 5c), consistent with detection of Ctcf at the Xa in some males. One interpretation of these data is that Ctcf binds Dxz4 at the Xa and Xi equally, but not detecting Ctcf at Dxz4 in all males even when it is readily detected in the same samples at a known Ctcf binding site within the H19 imprinted control region [47,48] suggests that binding of Ctcf to Dxz4 varies. This result could reflect subtle differences in CpG methylation (compare the two male bisulfite profiles in Figure 4b), strain or cell-type differences. Nevertheless, these observations are consistent with the differences we report above for Dxz4 chromatin organization at the Xa and Xi between mouse and primates. Notably, the association of Ctcf within the VNTR region means that although the array itself is relatively small, the potential Ctcf occupancy is higher than one per repeat monomer.

As mentioned above, the unique sequence (Ds-TR) located immediately distal to the large inverted satellite repeat (Figure 5a; Additional file 1) is characterized by DNaseI hypersensitivity and Ctcf binding (Additional file 6). Ctcf ChIP-PCR confirmed association with this sequence in males and females (Figure 5b), and as anticipated given the CpG hypomethylation (Figure 4b), the region was characterized by H3K4me2. To determine whether Ctcf at Ds-TR is associated with the Xa alone or with Xa and Xi, we used informative BL6 and cast SNPs to assign Ctcf ChIP-Seq reads to their X chromosome of origin. Unlike Dxz4, Ctcf at Ds-TR was biased toward the Xa but could also bind the Xi to a lower extent (Figure 5c).

### Conservation of a large tandem repeat downstream of *PLS3 *in mammals

**Figure 6:**
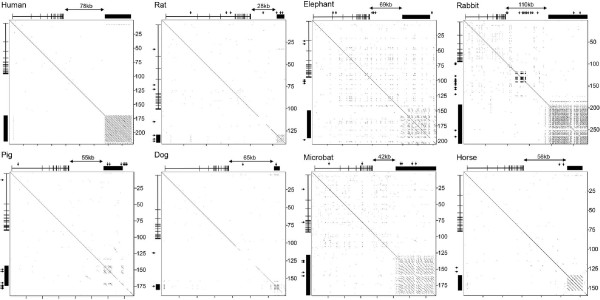
**Identification of a tandem repeat downstream of *PLS3 *in eight different mammals**. Pairwise alignment of genomic DNA sequence encompassing and extending downstream of *PLS3 *for each mammal (labeled above each plot). The structure and location of *PLS3 *is indicated on the top and left edge of each alignment. Distance in kilobases is indicated to the right of each plot. The distance between the 3' end of *PLS3 *and the downstream tandem repeat is highlighted above each plot. The extent of the tandem repeat is highlighted by the black bar above and to the left of each plot. Arrows pointing down from the top or rightward from the left edge indicate gaps in the genome assembly.

Thus far we have shown that, as in primates [14,17,22,30], a large tandem repeat is present downstream of *Pls3 *on the mouse X chromosome despite extensive shuffling of the locations of genes from the same interval (Figure 1a). We sought to determine whether a tandem repeat was present downstream of *PLS3 *in a diverse set of mammals for which genome assemblies were sufficiently complete. Pairwise alignment of genomic sequence distal to *PLS3 *was performed for seven different mammals. Each revealed the presence of a tandem repeat within 28 to 110 kb of the 3' end of *PLS3 *(Figure 6).

### Conservation of the CTCF binding sequence at DXZ4

**Figure 7:**
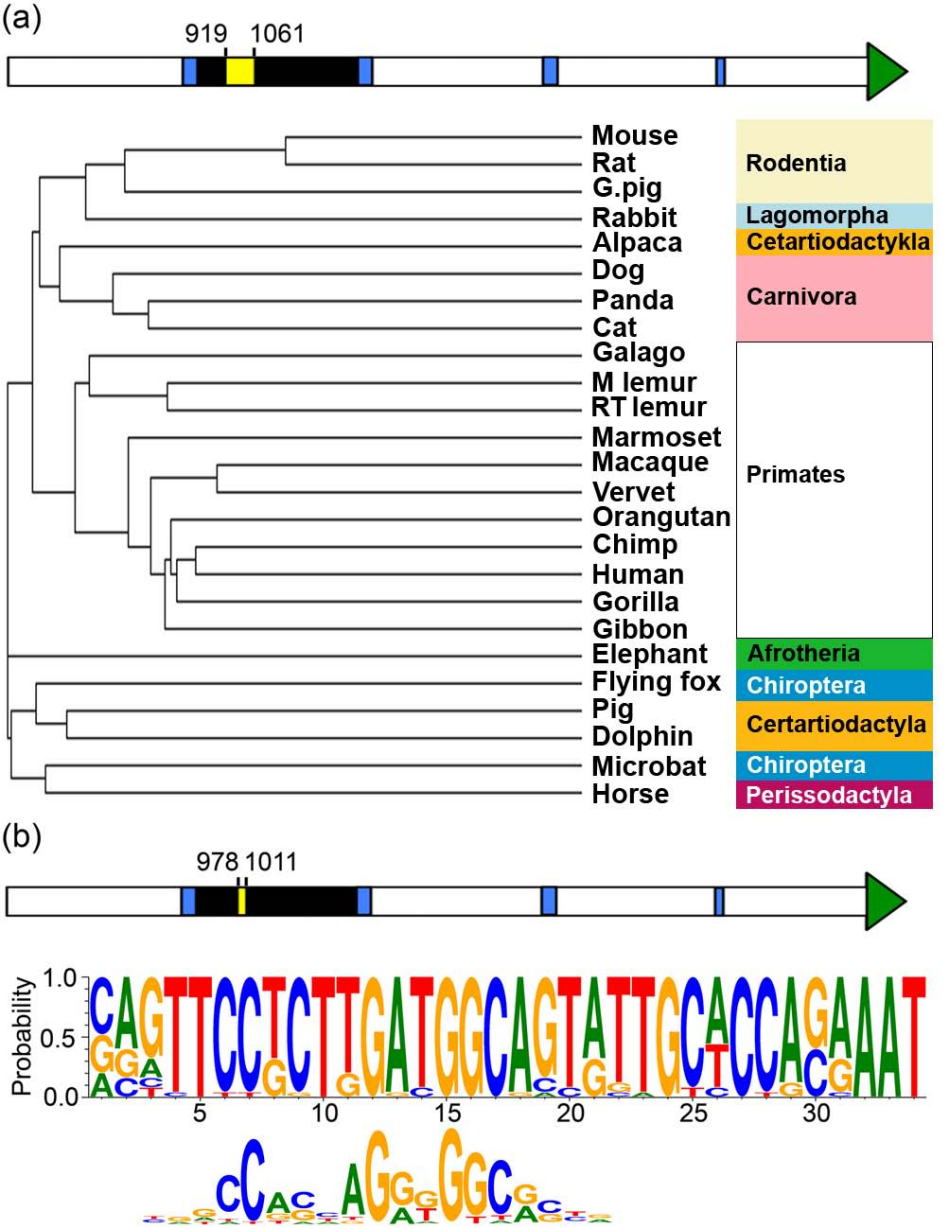
**Identification of a conserved DNA sequence element with homology to a CTCF consensus sequence in mammalian DXZ4**. **(a) **Schematic representation of a mouse Dxz4 monomer. The green arrowhead indicates the spliced exon. The blue vertical bars indicate repeat-masked sequence. The black bar represents the VNTR. The yellow box within the VNTR (bases 919 to 1,061) represents the conserved Dxz4 sequence. This sequence was used to align to the corresponding sequences from the mammals listed to generate the cladogram. The tree image was generated with MUSCLE version 3.8 [72] and ClustalW2 [73]. Classification of the groups is given to the right. **(b) **Schematic representation of a mouse Dxz4 monomer as above. The yellow box within the VNTR (bases 978 to 1,011) represents the DNA sequence that contains nucleotides invariable in all mammalian DXZ4 sequences assessed. This 34-bp sequence from each mammal was used to generate the position weight matrix through WebLogo [55]. Beneath the matrix is a previously determined Ctcf consensus sequence that is adapted from Martin *et al. *[47]. Note that the position weight matrix is the reverse complement of that shown in the referenced manuscript.

Previously we have shown that a region encompassing the CTCF binding site is conserved in primates, but outside of this interval divergence of the sequence of DXZ4 and size of the individual tandem repeat unit increases substantially with distance down the primate tree [30]. Focusing only on this region, we identified 74% nucleotide identity over 100 bp between human DXZ4 and the VNTR region within each mouse Dxz4 monomer. Similar levels of nucleotide identity over the same interval were identified within the tandem repeat DNAs shown in Figure 6. We used this interval to extract homologous DNA sequence entries from 25 different mammals before aligning all of the sequences. Most of the mammals examined formed clades corresponding to their respective orders and suborders, such as the primates, which all branch from a single node (Figure 7a). These data support evolution of DXZ4 from a common ancestor in a manner analogous to that of coding sequences. Close examination of the DNA sequence alignment revealed a subregion of the conserved DNA sequence in which several nucleotides were identical in all 25 mammals. A 34-bp sequence encompassing all invariable nucleotides was extracted from each sequence and used to generate a position weight matrix [55] that clearly revealed the nonrandom nature of this sequence (Figure 7b). Given that this sequence is entirely contained within the region assessed by PCR in primate CTCF ChIP [22,30], mouse Ctcf ChIP (Figure 5b), and mouse Ctcf ChIP-Seq (Figure 5c), the position weight matrix sequence was compared with a previously defined Ctcf consensus sequence [47], and as can be seen in Figure 7b, the most conserved DXZ4 sequence in all mammals examined was a good match to this consensus.

The Ctcf match to the conserved sequence only accounts for bases 3 to 21, yet conservation of DNA sequence across the diverse group of mammals extends for an additional 13 bp. It is conceivable that this extended conservation reflects retention of an additional binding motif(s) for other DNA binding protein(s). To explore this possibility, the consensus sequence was compared to motifs in JASPAR [56]. Two motifs showed good matches to this region. The first is a 9 out of 10 base match to the recently determined mouse consensus for the CCAAT/enhancer-binding protein alpha (Cebpa) [57], whereas the second is a match (9 out of 9) for the human consensus for ETS-domain protein 4 (ELK4) [58] (Additional file 7). Cebpa is an essential basic-leucine zipper DNA binding protein that performs essential roles in the development of myeloid cells [59] and in liver function [60]. ELK4 is a ubiquitous serum response factor accessory protein [61] that is found at many locations in the genome [62]. Whether either protein binds to Dxz4 has yet to be determined, but given the broad cross-species conservation of the DNA sequence and good matches with each DNA binding consensus sequence [57,58], both are candidates worthy of further investigation.

## Conclusions

Comparative genomics is a powerful means of uncovering important functional DNA elements through DNA sequence conservation [63], but identification of mouse Dxz4 was initially discovered not through primary DNA sequence conservation but instead through conservation of DNA sequence organization within a syntenic region of the mouse genome. This work led to the subsequent identification of DXZ4 in a diverse group of distantly related mammals. DNA sequence comparisons revealed a highly conserved region within each DXZ4 monomer that corresponds to the CTCF binding motif that is bound by CTCF in all mammals tested thus far. Furthermore, the highly conserved sequence immediately adjacent to the Ctcf consensus site suggests a second DNA binding protein may associate alongside Ctcf. Therefore, on the basis of conservation, several features of DXZ4 appear to have functional importance in eutherian mammals: CTCF binding, tandem-repeat organization, expression, and location downstream of *PLS3*.

In primates CTCF association with DXZ4 is almost exclusively Xi-specific [22,30], yet the analysis of mouse Dxz4 we report here suggests that its chromosome specificity is not as clearly defined; it apparent binds to both the Xa and the Xi to varying degrees. Primates and mouse appear to differ in several other aspects of DXZ4. First, primate DXZ4 is composed of a large number of tandem repeat units in which adjacent repeat monomers share very high DNA sequence identity and length [17,30]. The same is not true of mouse Dxz4. The tandem array is small in comparison, and individual repeat monomers display pronounced sequence variation and the presence of an internal VNTR. Perhaps near-identical sequence composition and monomer size are a prerequisite for expansion, such as the observed complex gene conversion mechanisms reported for minisatellites [64] or through alternative processes such as intrachromatid recombination or unequal exchange [65]. Second, DXZ4 DNA sequence is GC-rich in primates [14,17,22,30] but not in mouse. Third, DXZ4 in humans contains a DNA sequence with inherent promoter activity in each monomer [22]. This sequence is not conserved in mouse and intrinsic promoter activity is not obvious within the Dxz4 monomers. Instead a promoter located to one side of Dxz4 drives transcription across the entire array, but tandem repeat units in several other mammals do show substantial DNA sequence homology to human DXZ4 beyond the CTCF binding region encompassing the promoter sequence. These include cat, dog, horse, elephant, dolphin, microbat, rabbit, and flying fox (data not shown), suggesting that these mammals will likely retain internal promoter activity negating the need for the external promoter. Fourth, although all DXZ4 examined is transcribed [17,22,30], at least some mouse Dxz4 is spliced, a feature not observed in primates. Finally, euchromatin is largely restricted to DXZ4 on the Xi in primates [22,30] yet H3K4me2 is a feature of Dxz4 on the Xa in mouse. One feature that is consistent between the mouse and primate macrosatellite is significantly higher incidence of CpG hypomethylation in females that we interpret as originating from the Xi. Compared to primates, however, the overall profile is more methylated in mouse relative to primates [14,22,30]. Conceivably, the hypermethylation of Dxz4 combined with lower overall GC content is accelerating mutation of CpG dinucleotides [66].

Collectively, these observations suggest that the functions performed by DXZ4 in primates are not all necessarily conserved in mouse. We hypothesize that primate DXZ4 has important but distinct roles on the Xa and Xi that both necessitate a large homogenous tandem array. On the Xa this role involves expression and packaging into heterochromatin. Given the extreme copy-number variation of DXZ4 [14,17], the macrosatellite could conceivably modulate the transcription of the adjacent *PLS3 *gene, which shows considerable variation in expression levels between individuals [67]. In contrast, on the Xi a euchromatic organization bound by CTCF is required. The fact that CTCF is central to mediating genome organization [68], and that, at least in humans, CTCF-bound DXZ4 mediates Xi-specific long-range intrachromosomal interactions with other Xi-specific CTCF-bound tandem repeats [28] suggests that DXZ4 performs a structural role on the Xi. Mouse Dxz4 may or may not perform either function, and the difference could contribute to some of the observed differences between the biology of the human and mouse X chromosome, such as the variable escape of *PLS3 *expression from the Xi in humans [33] but not in mouse [34]. The distinct differences between DXZ4 and Dxz4 suggest that, if Dxz4 performs a similar function, it has evolved alternative strategies in order to do so. Nevertheless, the evolutionarily constrained association of CTCF/Ctcf with mammalian DXZ4 appears central even if conservation of function is not.

## Materials and methods

### Cells

Mouse male fibroblast cell line NIH/3T3 (CRL-1658) and female fibroblast cell line Balb/3T3 (CCL-163) were obtained from ATCC. Mouse female fibroblast cell line BC06 (hybrid C57BL/6J X castaneous) was obtained from Laura Carrel. Male and female CD-1 and C57BL/6J mouse embryonic fibroblasts were derived by standard techniques [69]. All cells were maintained in Dulbecco's modified Eagle's medium containing 10% fetal bovine serum supplemented with 1× nonessential amino acids, 2 mM L-glutamine, 100 U/ml penicillin, and 0.1 mg/ml streptomycin. All medium components were obtained from Invitrogen (Life Technologies Corp, Grand Island, NY, USA); NIH/3T3 cells were cultured in media containing Hyclone bovine calf serum (Thermo Scientific, Rockford, IL, USA) in place of fetal bovine serum.

### Bisulfite modification of DNA, cloning and sequencing

Genomic DNA was isolated from primary cells with the NucleoSpin Tissue kit (Machery-Nagel, Bethlehem, PA, USA). Genomic DNA was isolated from mouse tail snips by standard techniques [69]. Unmethylated cytosines were converted to uracil with the EpiTect bisulfite modification kit (Qiagen, Valencia, CA, USA). Bisulfite-modified DNA was used as a template for PCR with OneTaq^®^ master mix (NEB, Ipswich, MA, USA) and the primers listed in Additional file 8. PCR products were cloned into pDrive TA vector (Qiagen), and positive clones sequenced (Eurofins MWG Operon, Huntsville, AL, USA) and analyzed with Sequencher 5.0 (Gene Codes Corp., Ann Arbor, MI, USA). Statistically significant differences in methylation between males and females were determined as follows. The percent methylation for individual clones (a single horizontal line in the profiles) was determined and the mean and standard deviation was calculated for the males and females. These were compared using the two-tailed *t*-test with differing variance as described previously for methylation profiles [70].

### RNA and extended DNA fiber FISH

Mouse Dxz4 fragments were PCR amplified and cloned into the TA vector pCR2.1 (Life Technologies Corp.) before sequence verification. Direct-labeled FISH probes were generated from Dxz4-pCR2.1-isolated DNA with SpectrumOrange™ or SpectrumGreen™ and a nick translation kit (Abbott Molecular, Abbott Park, IL, USA). Probes were heat inactivated at 68°C for 10 minutes before ethanol precipitation and resuspension in Hybrisol VII (MP Biomedicals, Santa Ana, CA, USA). RNA FISH was performed on cells grown directly on microscope slides. Cells were rinsed with 1× phosphate-buffered saline (PBS) before being fixed and extracted for 10 minutes at room temperature in 3.7% formaldehyde, 0.1% Triton X-100 in 1× PBS. Slides were rinsed twice in 1× PBS before dehydration for 3 minutes in 70% and 100% ethanol before being air-dried. Probes were denatured in a thermal cycler at 72°C for 10 minutes before the temperature was reduced to 37°C, at which point the probe was applied directly to the slide, sealed under a cover glass, and hybridized overnight at 37°C. Cover slips were removed and the samples washed twice at room temperature for 2 minutes each in 50% formamide/2 SSC, once for 3 minutes at 37°C in 50% formamide/2× SSC, and once for 3 minutes at 37°C in 2× SSC before addition of ProLong^®^ Gold antifade reagent supplemented with DAPI (Life Technologies Corp.). Mouse extended DNA fibers were prepared and FISH performed essentially as previously described [17]. Images were either collected with a Zeiss Axiovert 200 M fitted with an AxioCam MRm and managed with AxioVision 4.4 software (Carl Zeiss microimaging) or collected with a DeltaVision pDV. Delta Vision images were deconvolved with softWoRx 3.7.0 (Applied Precision, Issaquah, WA, USA) and compiled with Adobe Photoshop CS2 (Adobe Systems).

### Standard and strand-specific cDNA preparation and PCR

Total RNA was extracted from cells with the NucleoSpin RNA II kit (Machery-Nagel). For standard RT-PCR, first-strand cDNA was prepared from 2 μg of total RNA with random hexamers with and without M-MuLV reverse transcriptase (RT) according to the manufacturer's instructions (NEB). cDNAs prepared with and without RT were used as templates for PCR with either OneTaq^®^ master mix (NEB) or HotStar Taq (Qiagen) with the primers listed in Additional file 8. PCR was performed using an initial denaturation of 10 minutes at 94°C, followed by 35 cycles of: 94°C for 30 seconds, 58°C for 30 seconds and 72°C for 30 seconds for all products of up to 750 bp, 1 minute for all products up to 1,250 bp and 1 minute 30 seconds for products up to 2 kb. The cycling was followed by 10 minutes at 72°C before holding at 15°C. Strand-specific cDNA was prepared as above except that first-strand cDNA was primed with 1.5 pmol of a specified oligonucleotide (Additional file 8) in place of random hexamers and an additional control that included RT but no oligonucleotide that is used to determine the background levels of cDNA synthesized in the absence of a gene-specific primer. Strand-specific cDNA was assessed by quantitative RT-PCR using the primers given (Additional file 8) with a SYBR-Green qPCR Mastermix (SABiosciences, Qiagen) on a CFX96 (Biorad, Hercules, CA, USA). PCR was performed using an initial 10-minute denaturing step at 95°C followed by 40 cycles of: 15 seconds at 95°C, 30 seconds at 60°C and 30 seconds at 72°C. The cycle was followed by a melt-curve. PCR was performed in triplicate and the transcript level determined relative to background.

### Promoter luciferase assay

DNA fragments initiating in and extending upstream of Dxz4 exon 1 were generated by PCR with Platinum^®^Taq (Life Technologies Corp.; 94°C for 2 minutes followed by 40 cycles of: 94°C for 30 seconds, 58°C for 30 seconds and 68°C for 1 minute 20 seconds for construct A or 68°C for 30 seconds for construct B) and cloned into pDrive (Qiagen). Inserts were verified by DNA sequencing before subcloning into the *Kpn*I and *Xho*I sites of pGL4.10[luc2] (Promega, Madison, WI, USA). The Dxz4-promoter pGL4.10[luc2] firefly luciferase reporter constructs were co-transfected in triplicate on two separate occasions with the *Renilla*-luciferase expression vector pGL4.74[hRluc/TK] (Promega) into NIH/3T3 cells by means of Lipofectamine 2000 (Life Technologies Corp.). Cells were assayed for luciferase activity on a Glomax-20/20 Luminometer (Promega) 72 hours after transfection with the dual-luciferase reporter assay system, according to the manufacturer's recommendations (Promega).

### ChIP and analysis

Standard ChIP was performed on mouse cells essentially as described previously [22] except that formaldehyde cross-linking was with 0.75% formaldehyde rather than 1.0%. Chromatin was sheared with a Bioruptor (Diagenode, Denville, NJ, USA) set at 8 cycles of 30 seconds on and 30 seconds off on high setting. Rabbit polyclonal antibodies used were all obtained from Millipore (Billerica, MA, USA) and included anti-H3K4me2 (07-030), anti-H3K27me3 (07-449), and anti-CTCF (07-729). ChIP was assessed by quantitative PCR using the primers given (Additional file 8) with a SYBR-Green qPCR Mastermix (SABiosciences, Qiagen) on a CFX96 (Biorad). PCR was performed using an initial 10-minute denaturing step at 95°C followed by 40 cycles of: 15 seconds at 95°C, 30 seconds at 60°C and 30 seconds at 72°C. The cycle was followed by a melt-curve. Standard curves were prepared by making a 1:5 serial dilution of the input for each ChIP. ChIP and mock (rabbit serum) samples were assessed in triplicate and the percentage of quantitative PCR product normalized and determined from the standard curve using Bio-Rad CFX Manager 2.1 software (Biorad). Each ChIP experiment and all PCR assessments were replicated on at least three independent occasions. Anti-Ctcf ChIP on mouse TSCs derived from a C57BL/6J × CAST/EiJ cross was combined with next-generation sequencing (100-bp paired-end reads) as described in detail elsewhere (Calabrese JM and Magnuson T, in preparation). Briefly, ChIP was performed on 10 to 40 × 10^6 ^feeder-free TSCs. Cells were crosslinked for 10 minutes at room temperature in 0.6% formaldehyde before quenching in 125mM glycine for 5 minutes. Cells were resuspended in 50 mM Tris-HCl pH 7.5, 140 mM NaCl, 1 mM EDTA, 1 mM EGTA, 0.1% Na-deoxycholate and 0.1% SDS. Cells were sonicated to generate fragments averaging 200 to 500 bp, cleared by centrifugation and resuspended at 20 × 10^6 ^cells/ml in the buffer above supplemented with 1% Triton-X100. ChIP was performed with 10 μg of antibody. Post-ChIP, three washes with the buffer used for the ChIP were performed, followed by a wash in the same buffer but with 500 mM NaCl, once with 20 mM Tris pH 8.0, 1 mM EDTA, 250 mM LiCl, 0.5% Na-deoxycholate and once with TE buffer. Chromatin was eluted for 15 minutes at 65°C in 50 mM Tris pH 8.0, 10 mM EDTA and 1% SDS. A ChIP-Seq library was prepared according to Illumina instructions using 10 to 200 ng of ChIP DNA and sequenced on Illumina's Genome Analyzer IIx or HiSeq2000 instrument. Ctcf ChIP-Seq data have been deposited with Gene Expression Omnibus and assigned the provisional accession number GSE40667. The DNA sequence of the mouse Dxz4 array was used to extract ChIP-Seq hits with homology to Dxz4. An approximately 232-bp DNA fragment spanning the putative mouse Dxz4 Ctcf binding site was amplified from C57BL/6J and castaneous genomic DNA isolated from tail snips. PCR was performed using HotStar Taq (Qiagen) with an initial denaturation of 10 minutes at 94°C, followed by 35 cycles of: 94°C for 30 seconds, 58°C for 30 seconds and 72°C for 30 seconds. The PCR product was cloned into pDrive, and for each DNA source over 100 clones were isolated and sequenced. Sequence variants specific to C57BL/6J and castaneous were then used to manually align with 100% sequence identity over a minimum of 30 bp to the Ctcf ChIP-Seq Dxz4 sequences and designated either C57BL/6J or castaneous. All SNP variants have been deposited with dbSNP. Details can be found in Additional file 9.

## Abbreviations

BAC: bacterial artificial chromosome; BL6: C57BL/6J mouse; bp: base pair; cast: castaneous mouse; cDNA: complementary DNA; CGI: CpG island; ChIP: chromatin immunoprecipitation; ChIP-Seq: ChIP combined with next generation sequencing; CTCF: CCCTC-binding factor; DD-CGI: CGI immediately downstream of the Dxz4 array; Ds-TR: downstream inverted tandem repeat; ENCODE: Encyclopedia of DNA Elements; FISH: fluorescence *in situ *hybridization; H3K4me2: histone H3 dimethylated at lysine 4; H3K4me3: histone H3 trimethylated at lysine 4; H3K27me3: histone H3 trimethylated at lysine 27; PBS: phosphate buffered saline; PCR: polymerase chain reaction; RT: reverse transcriptase; TSC: trophoblast stem cell; VNTR: variable number tandem repeat; Xa: active X chromosome; XCI: X-chromosome inactivation; Xi: inactive X chromosome; Xist: X-inactive specific transcript.

## Competing interests

The authors declare that they have no competing interests.

## Authors' contributions

BPC conceived the study, analyzed and interpreted data, performed experiments, and wrote the manuscript. AHH performed experiments and analyzed the data. MC performed Ctcf ChIP-Seq and analyzed the data. CRM and DT carried out experiments. All authors reviewed and contributed to the manuscript.

## Supplementary Material

Additional file 1 **Genomic organization and expression of the downstream tandem repeat**. The pair-wise alignment and repeat content of the Ds-TR as well as expression as demonstrated by RT-PCR. Click here for file

Additional file 2 **X chromosome tandem repeat survey**. Table summarizing large tandem repeat elements along the mouse X chromosome. Click here for file

Additional file 3 **Mouse BAC clones encompassing Dxz4**. BAC clones that completely span the mouse Dxz4 tandem repeat. Click here for file

Additional file 4 **Assessing Dxz4 for promoter activity**. Assessment of mouse Dxz4 for internal promoter activity. Click here for file

Additional file 5 **Dxz4 exon1 to tandem repeat monomer RT-PCR**. The presence of primary transcript bridging exon 1 of Dxz4 into the tandem repeat. Click here for file

Additional file 6 **CpG methylation relative to Ctcf and DNaseI hypersensitivity**. The location of a Ctcf and DNaseI peak relative to CpG methylation immediately adjacent to the Ds-TR. Click here for file

Additional file 7 **Motif alignments to Dxz4 conserved region**. Alignment of the Dxz4 conserved region with DNA binding protein motifs in JASPAR. Click here for file

Additional file 8 **Table listing all oligonucleotides used in this study**. Click here for file

Additional file 9 **Dxz4 SNP data**. List of SNPs identified in proximity to the Dxz4 Ctcf site in BL6 and cast DNA that were used to assign Ctcf ChIP-Seq fragments to the BL6 or cast chromosome in Figure 5c. Click here for file
