# Supplementary material for: The mouse DXZ4 homolog retains Ctcf binding and proximity to Pls3 despite substantial organizational differences compared to the primate macrosatellite
Source: Genome Biol. 2012 Aug 20;13(8):R70. doi: 10.1186/gb-2012-13-8-r70 (PMC3491370; doi:10.1186/gb-2012-13-8-r70)

Additional file 1: Genomic organization and expression of the downstream tandem repeat.

(a) Pairwise alignment of the tandem repeat genomic interval (72.89–72.92 Mb, mm9). Sequence homology is shown in blue with inverted homology in yellow pseudocoloured to avoid red-green. Above the alignment are annotated the location and where appropriate the orientation of repetitive elements. Distance in kilobases is given on the left and bottom edge. (b) Expression of the tandem repeat as determined by reverse transcription PCR. Images are ethidium bromide-stained agarose gels from male (NIH/3T3) and female (Balb3T3) cDNA. The expected size is indicated to the left. Samples include water (W), and total RNA treated with (+RT) and without (-RT) reverse transcriptase. The relative locations of the two PCRs are indicated by the "A" and "B" labelled left pointing arrows to the right of the pairwise alignment in (a).

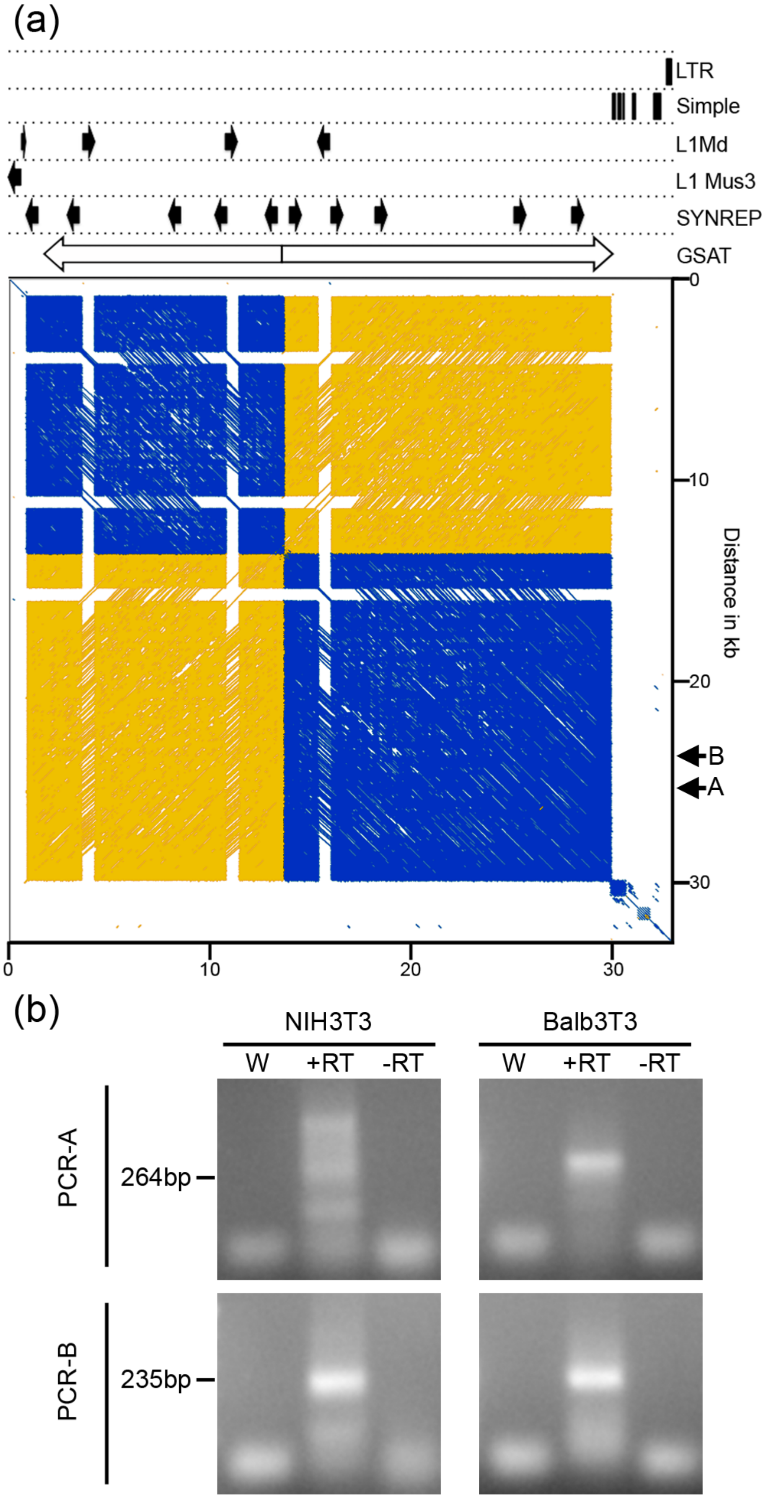

Supplement: Additional file 1 — Genomic organization and expression of the downstream tandem repeat. The pair-wise alignment and repeat content of the Ds-TR as well as expression as demonstrated by RT-PCR. [file gb-2012-13-8-r70-S1.PDF]
