# Supplementary material for: The mouse DXZ4 homolog retains Ctcf binding and proximity to Pls3 despite substantial organizational differences compared to the primate macrosatellite
Source: Genome Biol. 2012 Aug 20;13(8):R70. doi: 10.1186/gb-2012-13-8-r70 (PMC3491370; doi:10.1186/gb-2012-13-8-r70)
Supplement: Additional file 2 — X chromosome tandem repeat survey. Table summarizing large tandem repeat elements along the mouse X chromosome. [file gb-2012-13-8-r70-S2.PDF]

Additional File 2: X Chromosome Tandem Repeat Survey.
Table summarizes the results of comparing 1 Mb intervals of the mouse X chromosome against a repeat masked version of the same sequence in order to identify other substantial >90% unique tandem repeats such as the mouse Dxz4 described here, in order to ascertain how frequent such tandem repeats are on the mouse X chromosome. Pair-wise alignments performed using YASS (Noe and Kucherov, 2005, *Nucleic Acids Research* **33**:W540-W543). The rows highlighted in blue correspond to known mouse X segmental duplications (She et al., 2008, *Nature Genetics* **49**:909-914). Dxz4 is highlighted in red. Coordinates correspond to genome build mm9.

Coordinates	Tandem Repeat	Comments
1-5,500,000	-	Pericentric segmental duplications
5,500,001-6,500,000	No	-
6,500,001-7,500,000	No	-
7,500,001-8,500,000	Yes	SSX Cancer-Testis Locus
8,500,001-9,500,000	Yes	X-Y Segmental duplication
9,500,001-10,500,000	Yes	Located between Syt15 and Srpx
10,500,001-11,500,000	Yes	Histone cluster 2
11,500,001-12,500,000	No	-
12,500,001-13,500,000	Yes	Segmental duplication and Usp9x 3' UTR
13,500,001-14,500,000	No	-
14,500,001-15,500,000	No	-
15,500,001-16,500,000	No	-
16,500,001-17,500,000	No	-
17,500,001-18,500,000	No	-
18,500,001-19,500,000	No	-
19,500,001-20,500,000	No	-
20,500,001-21,500,000	Yes	Agtr2 upstream
21,500,001-22,500,000	No	-
22,500,001-23,500,000	No	-
23,500,001-24,500,000	Yes	Extensive segmental duplication
24,500,001-25,500,000	Yes	Extensive segmental duplication
25,500,001-26,500,000	Yes	Extensive segmental duplication
26,500,001-27,500,000	Yes	Extensive segmental duplication
27,500,001-28,500,000	Yes	Extensive segmental duplication
28,500,001-29,500,000	Yes	Extensive segmental duplication
29,500,001-30,500,000	Yes	Extensive segmental duplication
30,500,001-31,500,000	Yes	Extensive segmental duplication
31,500,001-32,500,000	Yes	Extensive segmental duplication
32,500,001-33,500,000	Yes	Extensive segmental duplication
33,500,001-34,500,000	Yes	Extensive segmental duplication
34,500,001-35,500,000	Yes	Extensive segmental duplication
35,500,001-36,500,000	No	-
36,500,001-37,500,000	No	-
37,500,001-38,500,000	No	-
38,500,001-39,500,000	No	-
39,500,001-40,500,000	No	-
40,500,001-41,500,000	No	-
41,500,001-42,500,000	No	-

42,500,001-43,500,000	No	-
43,500,001-44,500,000	Yes	Segmental duplication
44,500,001-45,500,000	No	-
45,500,001-46,500,000	No	-
46,500,001-47,500,000	No	-
47,500,001-48,500,000	Yes	Olfactory receptor gene Olfr1324
48,500,001-49,500,000	No	-
49,500,001-50,500,000	No	-
50,500,001-51,500,000	Yes	Extensive segmental duplication
51,500,001-52,500,000	Yes	Extensive segmental duplication
52,500,001-53,500,000	Yes	Extensive segmental duplication
53,500,001-54,500,000	Yes	Extensive segmental duplication
54,500,001-55,500,000	No	-
55,500,001-56,500,000	No	-
56,500,001-57,500,000	No	-
57,500,001-58,500,000	No	-
58,500,001-59,500,000	No	-
59,500,001-60,500,000	Yes	Extensive segmental duplication
60,500,001-61,500,000	No	-
61,500,001-62,500,000	No	-
62,500,001-63,500,000	No	-
63,500,001-64,500,000	Yes	miRNA Cluster
64,500,001-65,500,000	Yes	Segmental duplication
65,500,001-66,500,000	No	-
66,500,001-67,500,000	No	-
67,500,001-68,500,000	Yes	Segmental duplication
68,500,001-69,500,000	No	-
69,500,001-70,500,000	No	-
70,500,001-71,500,000	No	-
71,500,001-72,500,000	Yes	Extensive segmental duplication
72,500,001-73,500,000	Yes	Dxz4
73,500,001-74,500,000	No	-
74,500,001-75,500,000	No	-
75,500,001-76,500,000	No	-
76,500,001-77,500,000	No	-
77,500,001-78,500,000	No	-
78,500,001-79,500,000	No	-
79,500,001-80,500,000	No	-
80,500,001-81,500,000	Yes	Segmental duplications in Dmd gene
81,500,001-82,500,000	No	-
82,500,001-83,500,000	No	-
83,500,001-84,500,000	No	-
84,500,001-85,500,000	No	-
85,500,001-86,500,000	No	-
86,500,001-87,500,000	No	-
87,500,001-88,500,000	No	-
88,500,001-89,500,000	Yes	Extensive segmental duplication
89,500,001-90,500,000	No	-
90,500,001-91,500,000	No	-

91,500,001-92,500,000	Yes	Segmental duplications
92,500,001-93,500,000	No	-
93,500,001-94,500,000	No	-
94,500,001-95,500,000	No	-
95,500,001-96,500,000	No	-
96,500,001-97,500,000	No	-
97,500,001-98,500,000	No	-
98,500,001-99,500,000	No	-
99,500,001-100,500,000	Yes	DMRT-like family C1c2 cluster
100,500,001-101,500,000	No	-
101,500,001-102,500,000	No	-
102,500,001-103,500,000	No	-
103,500,001-104,500,000	Yes	Segmental duplication
104,500,001-105,500,000	No	-
105,500,001-106,500,000	No	-
106,500,001-107,500,000	No	-
107,500,001-108,500,000	No	-
108,500,001-109,500,000	No	-
109,500,001-110,500,000	No	-
110,500,001-111,500,000	No	-
111,500,001-112,500,000	No	-
112,500,001-113,500,000	No	-
113,500,001-114,500,000	No	-
114,500,001-115,500,000	No	-
115,500,001-116,500,000	No	-
116,500,001-117,500,000	No	-
117,500,001-118,500,000	No	-
118,500,001-119,500,000	No	-
119,500,001-120,500,000	Yes	Extensive segmental duplication
120,500,001-121,500,000	Yes	Extensive segmental duplication
121,500,001-122,500,000	Yes	Extensive segmental duplication
122,500,001-123,500,000	Yes	Extensive segmental duplication
123,500,001-124,500,000	No	-
124,500,001-125,500,000	No	-
125,500,001-126,500,000	No	-
126,500,001-127,500,000	No	-
127,500,001-128,500,000	No	-
128,500,001-129,500,000	No	-
129,500,001-130,500,000	No	-
130,500,001-131,500,000	No	-
131,500,001-132,500,000	Yes	Extensive segmental duplication
132,500,001-133,500,000	Yes	Segmental duplication
133,500,001-134,500,000	No	-
134,500,001-135,500,000	No	-
135,500,001-136,500,000	No	-
136,500,001-137,500,000	No	-
137,500,001-138,500,000	No	-
138,500,001-139,500,000	No	-
139,500,001-140,500,000	No	-

140,500,001-141,500,000	No	-
141,500,001-142,500,000	No	-
142,500,001-143,500,000	No	-
143,500,001-144,500,000	Yes	Extensive segmental duplication
144,500,001-145,500,000	Yes	Extensive segmental duplication
145,500,001-146,500,000	Yes	Extensive segmental duplication
146,500,001-147,500,000	Yes	Extensive segmental duplication
147,500,001-148,500,000	No	-
148,500,001-149,500,000	No	-
149,500,001-150,500,000	Yes	Spermatogenesis cluster
150,500,001-151,500,000	Yes	Mage cancer-testis locus
151,500,001-152,500,000	No	-
152,500,001-153,500,000	No	-
153,500,001-154,500,000	No	-
154,500,001-155,500,000	No	-
155,500,001-156,500,000	No	-
156,500,001-157,500,000	No	-
157,500,001-158,500,000	Yes	In Scml2 gene
158,500,001-159,500,000	No	-
159,500,001-160,500,000	No	-
160,500,001-161,500,000	No	-
161,500,001-162,500,000	No	-
162,500,001-163,500,000	No	-
163,500,001-164,500,000	No	-
164,500,001-165,500,000	No	-
165,500,001-166,500,000	Yes	Subtelomeric Region
