# Supplementary material for: The mouse DXZ4 homolog retains Ctcf binding and proximity to Pls3 despite substantial organizational differences compared to the primate macrosatellite
Source: Genome Biol. 2012 Aug 20;13(8):R70. doi: 10.1186/gb-2012-13-8-r70 (PMC3491370; doi:10.1186/gb-2012-13-8-r70)

Additional file 3: Mouse BAC Clones encompassing Dxz4.

Image extracted from the UCSC Genome Browser (http://genome.ucsc.edu/) showing the BAC end pair annotation on mouse genome build mm9 for the X chromosome coordinates 72,960,872-73,020,549. BAC clones are listed to the left, and those BACs that encompass the mouse Dxz4 array are highlighted by the red arrows to the left of the image. The Dxz4 array is highlighted in yellow and indicated by the labeled double-headed arrow at the bottom of the image.

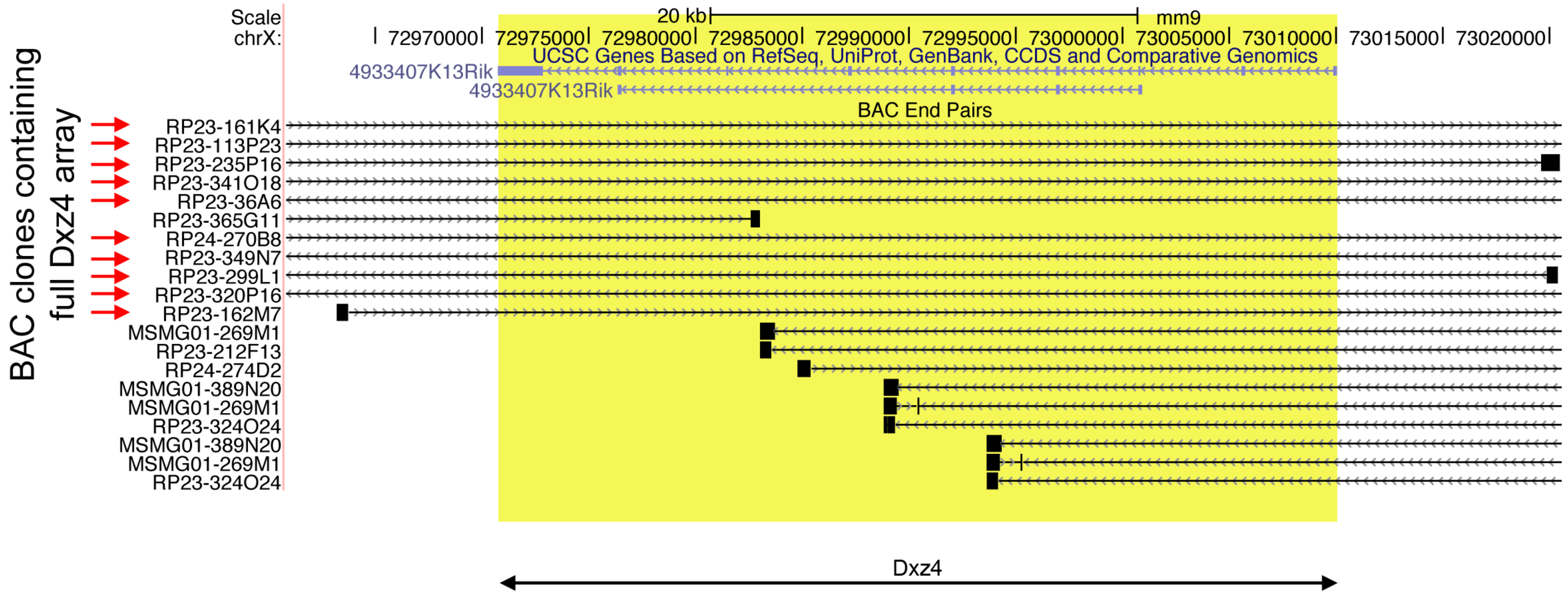

Supplement: Additional file 3 — Mouse BAC clones encompassing Dxz4. BAC clones that completely span the mouse Dxz4 tandem repeat. [file gb-2012-13-8-r70-S3.PDF]
