# Supplementary material for: The mouse DXZ4 homolog retains Ctcf binding and proximity to Pls3 despite substantial organizational differences compared to the primate macrosatellite
Source: Genome Biol. 2012 Aug 20;13(8):R70. doi: 10.1186/gb-2012-13-8-r70 (PMC3491370; doi:10.1186/gb-2012-13-8-r70)

Additional file 4: Assessing Dxz4 for promoter activity.

(a) Schematic map of a Dxz4 monomer represented by the white right-facing arrow. The thick black vertical lines within the arrow represent the simple repeats. The blue boxed area is the internal VNTR. Beneath the monomer are two orange rectangles that indicate the region cloned into the pGL4.10 vector. (b) Promoter activity for two independent fragments A (A-1 and A-2) and B (B-1 and B-2) relative to empty vector pGL4.10 and the mouse Dxz4 promoter construct B. Data shows fold activity for samples transfected in triplicate and measured in duplicate. Error bars show standard deviation.

a

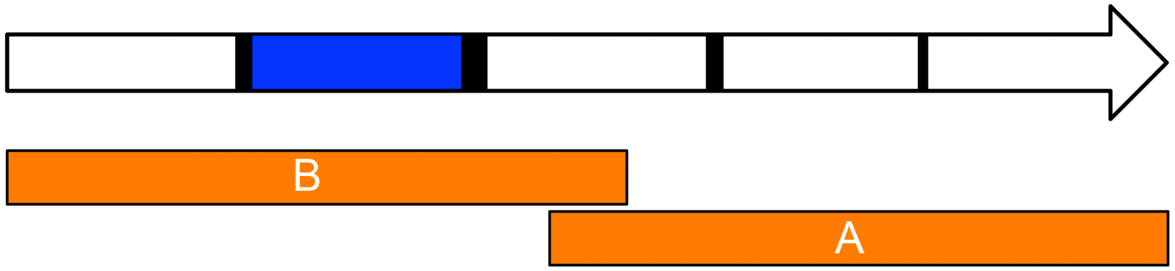

b

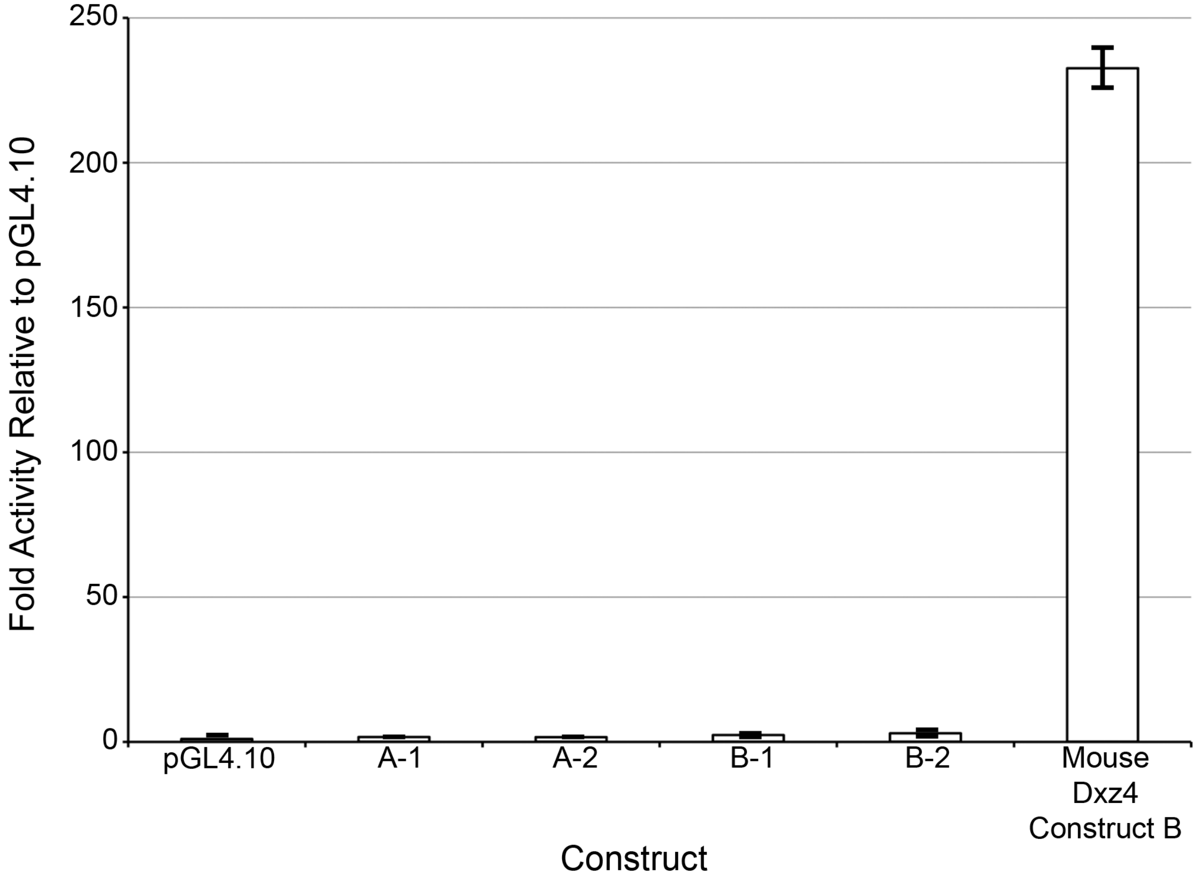

Supplement: Additional file 4 — Assessing Dxz4 for promoter activity. Assessment of mouse Dxz4 for internal promoter activity. [file gb-2012-13-8-r70-S4.PDF]
