# Supplementary material for: The mouse DXZ4 homolog retains Ctcf binding and proximity to Pls3 despite substantial organizational differences compared to the primate macrosatellite
Source: Genome Biol. 2012 Aug 20;13(8):R70. doi: 10.1186/gb-2012-13-8-r70 (PMC3491370; doi:10.1186/gb-2012-13-8-r70)

Additional file 5: Dxz4 exon 1 to tandem repeat monomer RT-PCR

(a) Schematic map of the interval from Dxz4 promoter to the first monomer of the tandem repeat (yellow block). The location of the minimal promoter is highlighted and red, exons 1 and 2 in blue and the interval between exon 1 and the beginning of the tandem repeat represented in grey. The ~2.5 kb region assessed by RT-PCR is represented beneath the map. (b) RT-PCR analysis for the region shown in part-a. Size of molecular weight marker fragments is indicated to the left, and samples assessed indicated above the gel. The PCR was performed using HotStar Taq (Qiagen) using the following cycle: 94°C for 15 minutes followed by 40 cycles of 94°C for 30 seconds, 58°C for 30 seconds and 72°C for 2 minutes 30 seconds. PCR used primers Dxz4-As-F1 with Dxz4-F9.

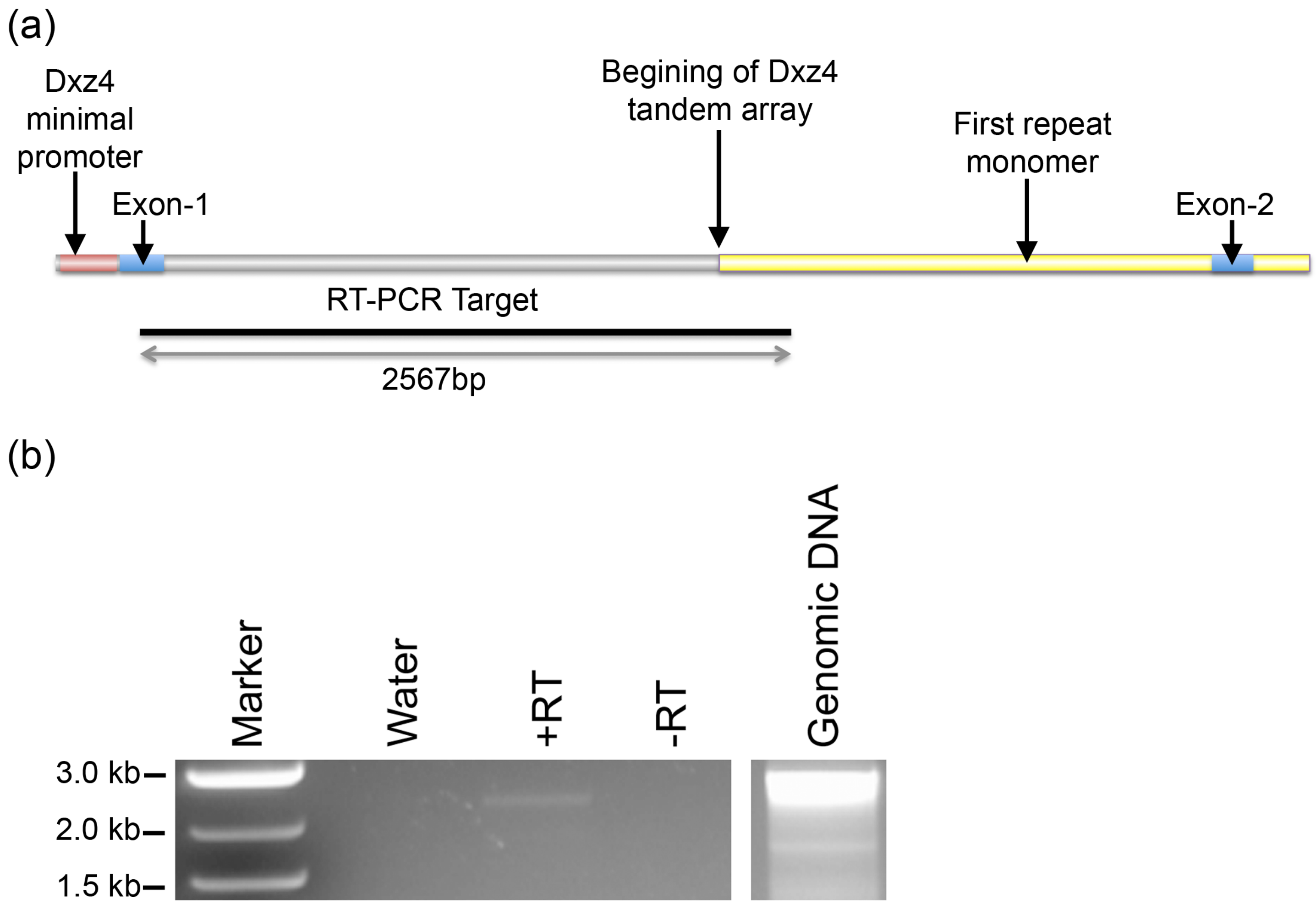

Supplement: Additional file 5 — Dxz4 exon1 to tandem repeat monomer RT-PCR. The presence of primary transcript bridging exon 1 of Dxz4 into the tandem repeat. [file gb-2012-13-8-r70-S5.PDF]
