# Supplementary material for: The mouse DXZ4 homolog retains Ctcf binding and proximity to Pls3 despite substantial organizational differences compared to the primate macrosatellite
Source: Genome Biol. 2012 Aug 20;13(8):R70. doi: 10.1186/gb-2012-13-8-r70 (PMC3491370; doi:10.1186/gb-2012-13-8-r70)

Additional file 6: CpG methylation relative to Ctcf and DNaseI hypersensitivity.
Locations of Ds-TR CpG methylation profiles relative to ChIP-seq data for Ctcf (Kim et al., 2007, *Cell* **128**: 1231-145) and DNaseI hypersensitivity (Sabo et al., 2006, *Nature Methods* **3**:511-518). Region shown corresponds to 72,917,882-72,920,900 of the mouse X chromosome (mm9).

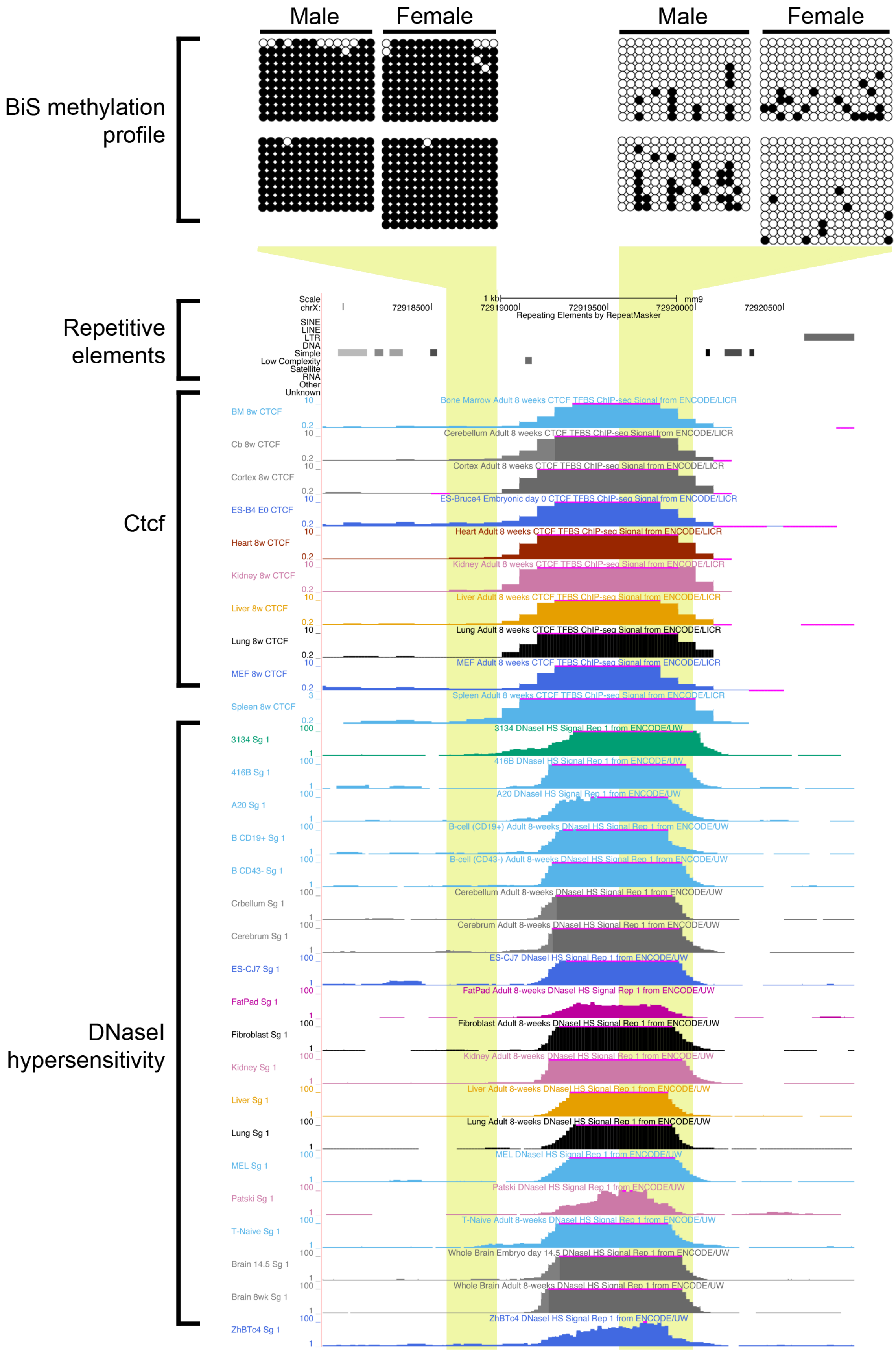

Supplement: Additional file 6 — CpG methylation relative to Ctcf and DNaseI hypersensitivity. The location of a Ctcf and DNaseI peak relative to CpG methylation immediately adjacent to the Ds-TR. [file gb-2012-13-8-r70-S6.PDF]
