# Supplementary material for: The mouse DXZ4 homolog retains Ctcf binding and proximity to Pls3 despite substantial organizational differences compared to the primate macrosatellite
Source: Genome Biol. 2012 Aug 20;13(8):R70. doi: 10.1186/gb-2012-13-8-r70 (PMC3491370; doi:10.1186/gb-2012-13-8-r70)

Additional file 7: Motif alignments to Dxz4 conserved region.

Figure shows alignments of motifs for DNA binding proteins relative to the Dxz4 ultra-conserved consensus sequence. Top alignment shows mouse Ctcf motif and bottom alignment shows human ELK4, both taken from JASPAR (http://jaspar.genereg.net/)(Bryne et al., 2008 Nucleic Acids Research:D102-6). The middle alignment shows mouse Cebpa adapted from Schmidt et al (Science 2010, 328:1036-1040).

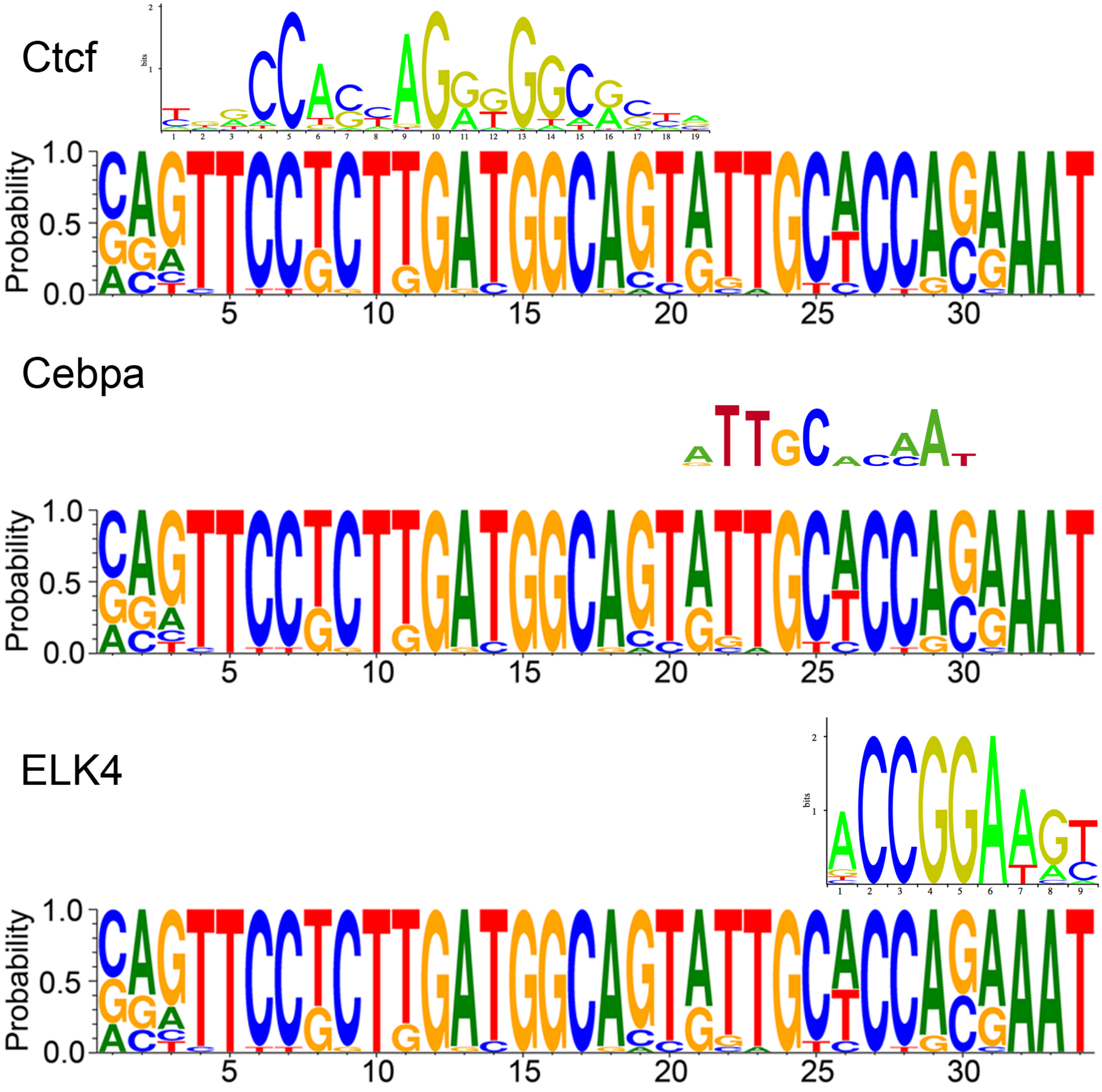

Supplement: Additional file 7 — Motif alignments to Dxz4 conserved region. Alignment of the Dxz4 conserved region with DNA binding protein motifs in JASPAR. [file gb-2012-13-8-r70-S7.PDF]
