# Supplementary material for: The mouse DXZ4 homolog retains Ctcf binding and proximity to Pls3 despite substantial organizational differences compared to the primate macrosatellite
Source: Genome Biol. 2012 Aug 20;13(8):R70. doi: 10.1186/gb-2012-13-8-r70 (PMC3491370; doi:10.1186/gb-2012-13-8-r70)
Supplement: Additional file 8 — Table listing all oligonucleotides used in this study. [file gb-2012-13-8-r70-S8.PDF]

Additional file 8: Table listing oligonucleotides used in this study.

Name	DNA Sequence	Primary Application
Dxz4-F1	GCTTAGTCTTCTTACCCAGG	RT-PCR unspliced transcript & FISH probes
Dxz4-R1	GTTAGTGAGCATGTGGAGAG	"
Dxz4-F2	TCTCTGCCTCTGGATCACC	"
Dxz4-R2	ATTCAGTGTGTGTCCCATGG	"
Dxz4-F3	AGAAAGAAACTCGGGTCTCC	"
Dxz4-R3	TGTCTCAGTATCTCAGGTAGC	"
Dxz4-F4	TGACTGAAAGCAGCTGACAG	"
Dxz4-R4	AGGAGTGTCTGTGATCAGTG	"
Dxz4-F5	TGCCTGAAAGCAGCTGAAAG	"
Dxz4-R5	TCTTAGGCACTACTAGCAGG	"
Dxz4-F6	AGTAGTGATGATGAGGGTGC	RT-PCR unspliced transcript, FISH probes & SS-RT primer
Dxz4-R6	TTCATCTCCCATTGGACTCG	"
Dxz4-F9	TGCCTGCTTGGATGTCAATC	RT-PCR Dxz4 exon 1 to tandem repeat
Dxz4-5-BiS-F1	GTTTGGTGGTATAGAATTTTAATG	BiS PCR - Dxz4 promoter
Dxz4-5-BiS-R2	TACTTAATCCAAACCAAACTATCC	"
Dxz4-BiS-F1	GTTTGGTGAATAGTGATTGGTAGG	BiS PCR - Dxz4 array
Dxz4-BiS-R1	TAATACTCAAACTCCATATTTATAAC	"
Dxz4-BiS DD-CGI-F2	AGGGTTTTTTAATATTGGAATG	BiS PCR - Dxz4 downstream CGI
Dxz4-BiS DD-CGI-R1	CATAAAATATCTTAACTACCCTTC	"
Dxz4-BiS-ds-F2	GAGGATGGTAAGATAGGGAG	BiS PCR - Dxz4 downstream tandem repeat
Dxz4-BiS-ds-R2	CTTCCTTCCTTATAACCTAACC	"
Dxz4-BiS-ds-F3	TGGTTTGTAGTTATATTATTTGG	"
Dxz4-BiS-ds-R3	CCAAAAACTCAAATCTACCCAC	"
Dxz4-As-F1	AAGAATGCAGAGTCAGGGAG	Dxz4 Exon 1-2 RT-PCR & exon 1 to tandem repeat
Dxz4-As-R1	CCTAGCCATATCAACCTGAC	"
Dxz4-As-F3	CTGAGAAACCAACCTGTGTG	Dxz4 Exon 2-3 RT-PCR
Dxz4-As-R3	ATGAGTTCCGGTGAATGCTG	"
Dxz4-As-F4	TTCTACCTAGACCTGGATAG	Dxz4 Exon 10-11 RT-PCR with Dxz4-As-R7
Dxz4-As-R5	TTAGCTCTAAATGGGTCCAC	Dxz4 Exon 1-9/10 RT-PCR with Dxz4-As-F1
Dxz4-As-F6	CAGCTTAGGTATAATGTAAAGGG	Dxz4 Exon 9-alt RT-PCR with Dxz4-As-R8
Dxz4-As-R6	CTCCTAACAGTTCTTCACAG	Dxz4 Exon 8-10 RT-PCR with Dxz4-As-F3
Dxz4-As-R7	GCTGCACACATCTTATACAG	Dxz4 Exon 10-11 RT-PCR with Dxz4-As-F4
Dxz4-As-R8	CCAGCTTAAGTGTCTCTGACC	Dxz4 Exon 8-9 RT-PCR with Dxz4-As-F3
Dxz4-F15	GGTACCTGGGAAAGGGATGAATGAAG	Promoter construct A
Dxz4-R15	CTCGAGCTTAGTTGGGAGCGAAGCAG	Promoter construct A and B
Dxz4-F17	GGTACCGGCCTGGTGGCACAGAACTC	Promoter construct B
Dxz4-F12	AACCCTGTGTAAATCTGGGC	RT-PCR of strand-specific cDNA
Dxz4-R12	TGGACCTGGAAACTTGAGAG	"
MsH19-F2	ATGCCTCAGTGGTCGATATG	H19 Ctcf ChIP qPCR
MsH19-R2	CTCTGGTTCAGTGTGTAAGG	"
qDxz4-Prom-F1	AGAGCGGTCTTTGTGCATCT	Dxz4 promoter H3K27me3 & H3K4me2 qChIP PCR
qDxz4-Prom-R1	AACTCACCAACAAGGCCAAG	"
qPls3-Prom-F1	GGGACTGGAACTCTGTTTGC	Pls3 promoter H3K27me3 & H3K4me2 qChIP PCR
qPls3-Prom-R1	CACGCCAAGCCTTGTAGATT	"
qDD-CGI-F1	AGCAGGAGCCGCTTCCAAG	DD-CGI H3K4me2 qChIP PCR
qDD-CGI-R2	GGCATGTTAGTGTCCCTATC	"
qDs-TR-F3	CCTGGGAACCGACTGATTTA	Ds-TR Ctcf & H3K4me2 qChIP PCR
qDs-TR-R3	AGCGTTAGGGCTGAGATGAG	"
Dxz4-F13	GTCGGCCACCATGTTAAGAG	Dxz4 Ctcf & H3K4me2 qChIP PCR
Dxz4-R13	CTCCATGTTTGTGGCTGGTG	"
Dxz4-ds-SO-F1	ATGAGGAACACACACTCTAG	Dxz4 downstream tandem repeat RT-PCR A
Dxz4-ds-SO-R1	CACATACCATCTTCTACAGTG	"
Dxz4-ds-SO-F2	GGTATATGTCCACAAACACTG	Dxz4 downstream tandem repeat RT-PCR B
Dxz4-ds-SO-R2	GTTCTCAAGTGTGCTTTTCTC	"
