# Supplementary material for: The mouse DXZ4 homolog retains Ctcf binding and proximity to Pls3 despite substantial organizational differences compared to the primate macrosatellite
Source: Genome Biol. 2012 Aug 20;13(8):R70. doi: 10.1186/gb-2012-13-8-r70 (PMC3491370; doi:10.1186/gb-2012-13-8-r70)
Supplement: Additional file 9 — Dxz4 SNP data. List of SNPs identified in proximity to the Dxz4 Ctcf site in BL6 and cast DNA that were used to assign Ctcf ChIP-Seq fragments to the BL6 or cast chromosome in Figure 5c. [file gb-2012-13-8-r70-S9.PDF]

Additional file 9: Dxz4 SNP data.

SNP data submitted to dbSNP:

```
TYPE:   CONT
HANDLE: CHADWICKLAB
NAME:   Brian Chadwick
FAX:    850 645 8447
TEL:    850 645 9279
EMAIL:  chadwick@bio.fsu.edu
LAB:    Chadwick Lab
INST:   Florida State University
ADDR:   3076, King Life Science Building, 319 Stadium Drive, Florida State University, Tallahassee, FL 32306-
4295, USA
||
TYPE:   METHOD
#added value to TYPE
HANDLE: CHADWICKLAB
ID:     Dxz4-Variants
METHOD_CLASS:  Sequence
SEQ_BOTH_STRANDS:
TEMPLATE_TYPE:
MULT_PCR_AMPLIFICATION:
MULT_CLONES_TESTED:
METHOD: The DNA sequence for these polymorphisms was obtained by PCR amplification from C56BL/6 or castaneous
genomic DNA, TA cloning followed by Sanger sequencing. Variants were confirmed in ChIP-Seq data from a
C56BL/6:castaneous sample.
||
TYPE:   SNPASSAY
HANDLE: CHADWICKLAB
BATCH:  2012A
MOLTYPE:        Genomic
METHOD: Dxz4-Variants
SAMPLESIZE:     8
ORGANISM:       Mus musculus
CITATION:
POPULATION:
COMMENT:
||
SNP:    IR-21
ACCESSION:
COMMENT:
        Forward PCR primer:GCCAAGAAGGTCTCAGGGAG
        Reverse PCR primer:GGCTCCATGTTTGTGGCTGG
        Chromosome- X
SAMPLESIZE:     8
LENGTH: ?
5'_FLANK:       CCTGGGCCAAGAAGGTCTCAGGGAG
OBSERVED:       C/T
3'_FLANK:       CCCCAGTCCCTCCTTGGCTGCCAGGAAAAGAGCGGGTCGGCCACCATGTTAAGAGAGGCGCAGGGATGTGCAGG
||

SNP:    IR-26
ACCESSION:
COMMENT:
        Forward PCR primer:GCCAAGAAGGTCTCAGGGAG
        Reverse PCR primer:GGCTCCATGTTTGTGGCTGG
        Chromosome- X
SAMPLESIZE:     8
LENGTH: ?
5'_FLANK:       GCCAAGAAGGTCTCAGGGAGCCCCC
OBSERVED:       A/C
3'_FLANK:       GTCCCTCCTTGGCTGCCAGGAAAAGAGCGGGTCGGCCACCATGTTAAGAGAGGCGCAGGGATGTGCAGGCGGAA
||

SNP:    IR-28
ACCESSION:
COMMENT:
        Forward PCR primer:GCCAAGAAGGTCTCAGGGAG
        Reverse PCR primer:GGCTCCATGTTTGTGGCTGG
        Chromosome- X
SAMPLESIZE:     8
LENGTH: ?
5'_FLANK:       GCCAAGAAGGTCTCAGGGAGCCCCCAG
OBSERVED:       T/C
3'_FLANK:
        CCCTCCTTGGCTGCCAGGAAAAGAGCGGGTCGGCCACCATGTTAAGAGAGGCGCAGGGATGTGCAGGCGGAAACCTCAAACCAATTCCGCTTGATGGCAGTAGA
GCACCAAGAAAGGCAACTGCCAGGCTGCTGGGCGCTCTCCCTCTGCCCTTCCAGAGGCAAAAAACGCCAACCTACAACCACCAGCCACAAACATGGAGCC
||
```

SNP: IR-30
ACCESSION:
COMMENT:
Forward PCR primer:GCCAAGAAGGTCTCAGGGAG
Reverse PCR primer:GGCTCCATGTTTGTGGCTGG
Chromosome- X
SAMPLESIZE: 8
LENGTH: ?
5'_FLANK: GCCAAGAAGGTCTCAGGGAGCCCCCAGTC
OBSERVED: C/T
3'_FLANK:
CTCCTTGGCTGCCAGGAAAAGAGCGGGTCGGCCACCATGTTAAGAGAGGCGCAGGGATGTGCAGGCGGAAACCTCAAACCAATTCCGCTTGATGGCAGTAGAGC
ACCAAGAAAGGCAACTGCCAGGCTGCTGGGCGCTCTCCCTCTGCCCTTCCAGAGGCAAAAAACGCCAACCTACAACCACCAGCCACAAACATGGAGCC
||

SNP: IR-32
ACCESSION:
COMMENT:
Forward PCR primer:GCCAAGAAGGTCTCAGGGAG
Reverse PCR primer:GGCTCCATGTTTGTGGCTGG
Chromosome- X
SAMPLESIZE: 8
LENGTH: ?
5'_FLANK: GCCAAGAAGGTCTCAGGGAGCCCCCAGTCCC
OBSERVED: T/C
3'_FLANK:
CCTTGGCTGCCAGGAAAAGAGCGGGTCGGCCACCATGTTAAGAGAGGCGCAGGGATGTGCAGGCGGAAACCTCAAACCAATTCCGCTTGATGGCAGTAGAGCAC
CAAGAAAGGCAACTGCCAGGCTGCTGGGCGCTCTCCCTCTGCCCTTCCAGAGGCAAAAAACGCCAACCTACAACCACCAGCCACAAACATGGAGCC
||

SNP: IR-40
ACCESSION:
COMMENT:
Forward PCR primer:GCCAAGAAGGTCTCAGGGAG
Reverse PCR primer:GGCTCCATGTTTGTGGCTGG
Chromosome- X
SAMPLESIZE: 8
LENGTH: ?
5'_FLANK: GCCAAGAAGGTCTCAGGGAGCCCCCAGTCCCTCCTTGGC
OBSERVED: T/C
3'_FLANK:
GCCAGGAAAAGAGCGGGTCGGCCACCATGTTAAGAGAGGCGCAGGGATGTGCAGGCGGAAACCTCAAACCAATTCCGCTTGATGGCAGTAGAGCACCAAGAAAG
GCAACTGCCAGGCTGCTGGGCGCTCTCCCTCTGCCCTTCCAGAGGCAAAAAACGCCAACCTACAACCACCAGCCACAAACATGGAGCC
||

SNP: IR-44
ACCESSION:
COMMENT:
Forward PCR primer:GCCAAGAAGGTCTCAGGGAG
Reverse PCR primer:GGCTCCATGTTTGTGGCTGG
Chromosome- X
SAMPLESIZE: 8
LENGTH: ?
5'_FLANK: GCCAAGAAGGTCTCAGGGAGCCCCCAGTCCCTCCTTGGCTGCC
OBSERVED: A/C
3'_FLANK:
GGAAAAGAGCGGGTCGGCCACCATGTTAAGAGAGGCGCAGGGATGTGCAGGCGGAAACCTCAAACCAATTCCGCTTGATGGCAGTAGAGCACCAAGAAAGGCAA
CTGCCAGGCTGCTGGGCGCTCTCCCTCTGCCCTTCCAGAGGCAAAAAACGCCAACCTACAACCACCAGCCACAAACATGGAGCC
||

SNP: IR-48
ACCESSION:
COMMENT:
Forward PCR primer:GCCAAGAAGGTCTCAGGGAG
Reverse PCR primer:GGCTCCATGTTTGTGGCTGG
Chromosome- X
SAMPLESIZE: 8
LENGTH: ?
5'_FLANK: GCCAAGAAGGTCTCAGGGAGCCCCCAGTCCCTCCTTGGCTGCCAGGA
OBSERVED: A/G
3'_FLANK:
AAGAGCGGGTCGGCCACCATGTTAAGAGAGGCGCAGGGATGTGCAGGCGGAAACCTCAAACCAATTCCGCTTGATGGCAGTAGAGCACCAAGAAAGGCAACTGC
CAGGCTGCTGGGCGCTCTCCCTCTGCCCTTCCAGAGGCAAAAAACGCCAACCTACAACCACCAGCCACAAACATGGAGCC
||

SNP: IR-51
ACCESSION:
COMMENT:
Forward PCR primer:GCCAAGAAGGTCTCAGGGAG
Reverse PCR primer:GGCTCCATGTTTGTGGCTGG

Chromosome- X
SAMPLESIZE: 8
LENGTH: ?
5'_FLANK: GCCAAGAAGGTCTCAGGGAGCCCCCAGTCCCTCCTTGGCTGCCAGGAAAA
OBSERVED: G/C
3'_FLANK:
AGCGGGTCGGCCACCATGTTAAGAGAGGCGCAGGGATGTGCAGGCGGAAACCTCAAACCAATTCCGCTTGATGGCAGTAGAGCACCAAGAAAGGCAACTGCCAG
GCTGCTGGGCGCTCTCCCTCTGCCCTTCCAGAGGCAAAAAACGCCAACCTACAACCACCAGCCACAAACATGGAGCC
||

SNP: IR-53
ACCESSION:
COMMENT:
Forward PCR primer:GCCAAGAAGGTCTCAGGGAG
Reverse PCR primer:GGCTCCATGTTTGTGGCTGG
Chromosome- X
SAMPLESIZE: 8
LENGTH: ?
5'_FLANK: GCCAAGAAGGTCTCAGGGAGCCCCCAGTCCCTCCTTGGCTGCCAGGAAAAGA
OBSERVED: G/A
3'_FLANK:
CGGGTCGGCCACCATGTTAAGAGAGGCGCAGGGATGTGCAGGCGGAAACCTCAAACCAATTCCGCTTGATGGCAGTAGAGCACCAAGAAAGGCAACTGCCAGGC
TGCTGGGCGCTCTCCCTCTGCCCTTCCAGAGGCAAAAAACGCCAACCTACAACCACCAGCCACAAACATGGAGCC
||

SNP: IR-55
ACCESSION:
COMMENT:
Forward PCR primer:GCCAAGAAGGTCTCAGGGAG
Reverse PCR primer:GGCTCCATGTTTGTGGCTGG
Chromosome- X
SAMPLESIZE: 8
LENGTH: ?
5'_FLANK: GCCAAGAAGGTCTCAGGGAGCCCCCAGTCCCTCCTTGGCTGCCAGGAAAAGAGC
OBSERVED: G/A
3'_FLANK:
GGTCGGCCACCATGTTAAGAGAGGCGCAGGGATGTGCAGGCGGAAACCTCAAACCAATTCCGCTTGATGGCAGTAGAGCACCAAGAAAGGCAACTGCCAGGCTG
CTGGGCGCTCTCCCTCTGCCCTTCCAGAGGCAAAAAACGCCAACCTACAACCACCAGCCACAAACATGGAGCC
||

SNP: IR-59
ACCESSION:
COMMENT:
Forward PCR primer:GCCAAGAAGGTCTCAGGGAG
Reverse PCR primer:GGCTCCATGTTTGTGGCTGG
Chromosome- X
SAMPLESIZE: 8
LENGTH: ?
5'_FLANK: GCCAAGAAGGTCTCAGGGAGCCCCCAGTCCCTCCTTGGCTGCCAGGAAAAGAGCGGGT
OBSERVED: C/T/G
3'_FLANK:
GGCCACCATGTTAAGAGAGGCGCAGGGATGTGCAGGCGGAAACCTCAAACCAATTCCGCTTGATGGCAGTAGAGCACCAAGAAAGGCAACTGCCAGGCTGCTGG
GCGCTCTCCCTCTGCCCTTCCAGAGGCAAAAAACGCCAACCTACAACCACCAGCCACAAACATGGAGCC
||

SNP: IR-61
ACCESSION:
COMMENT:
Forward PCR primer:GCCAAGAAGGTCTCAGGGAG
Reverse PCR primer:GGCTCCATGTTTGTGGCTGG
Chromosome- X
SAMPLESIZE: 8
LENGTH: ?
5'_FLANK: GCCAAGAAGGTCTCAGGGAGCCCCCAGTCCCTCCTTGGCTGCCAGGAAAAGAGCGGGTCG
OBSERVED: G/A
3'_FLANK:
CCACCATGTTAAGAGAGGCGCAGGGATGTGCAGGCGGAAACCTCAAACCAATTCCGCTTGATGGCAGTAGAGCACCAAGAAAGGCAACTGCCAGGCTGCTGGGC
GCTCTCCCTCTGCCCTTCCAGAGGCAAAAAACGCCAACCTACAACCACCAGCCACAAACATGGAGCC
||

SNP: IR-64
ACCESSION:
COMMENT:
Forward PCR primer:GCCAAGAAGGTCTCAGGGAG
Reverse PCR primer:GGCTCCATGTTTGTGGCTGG
Chromosome- X
SAMPLESIZE: 8
LENGTH: ?
5'_FLANK: GCCAAGAAGGTCTCAGGGAGCCCCCAGTCCCTCCTTGGCTGCCAGGAAAAGAGCGGGTCGGCC
OBSERVED: A/G

```
3'_FLANK:
        CCATGTTAAGAGAGGCGCAGGGATGTGCAGGCGGAAACCTCAAACCAATTCCGCTTGATGGCAGTAGAGCACCAAGAAAGGCAACTGCCAGGCTGCTGGGCGCT
CTCCCTCTGCCCTTCCAGAGGCAAAAAACGCCAACCTACAACCACCAGCCACAAACATGGAGCC
||

SNP:    IR-72
ACCESSION:
COMMENT:
        Forward PCR primer:GCCAAGAAGGTCTCAGGGAG
        Reverse PCR primer:GGCTCCATGTTTGTGGCTGG
        Chromosome- X
SAMPLESIZE:    8
LENGTH: ?
5'_FLANK:      GCCAAGAAGGTCTCAGGGAGCCCCCAGTCCCTCCTTGGCTGCCAGGAAAAGAGCGGGTCGGCCACCATGTT
OBSERVED:      A/C/T
3'_FLANK:
        AGAGAGGCGCAGGGATGTGCAGGCGGAAACCTCAAACCAATTCCGCTTGATGGCAGTAGAGCACCAAGAAAGGCAACTGCCAGGCTGCTGGGCGCTCTCCCTCT
GCCCTTCCAGAGGCAAAAAACGCCAACCTACAACCACCAGCCACAAACATGGAGCC
||

SNP:    IR-75
ACCESSION:
COMMENT:
        Forward PCR primer:GCCAAGAAGGTCTCAGGGAG
        Reverse PCR primer:GGCTCCATGTTTGTGGCTGG
        Chromosome- X
SAMPLESIZE:    8
LENGTH: ?
5'_FLANK:      GCCAAGAAGGTCTCAGGGAGCCCCCAGTCCCTCCTTGGCTGCCAGGAAAAGAGCGGGTCGGCCACCATGTTAAG
OBSERVED:      A/C/G
3'_FLANK:
        GAGGCGCAGGGATGTGCAGGCGGAAACCTCAAACCAATTCCGCTTGATGGCAGTAGAGCACCAAGAAAGGCAACTGCCAGGCTGCTGGGCGCTCTCCCTCTGCC
CTTCCAGAGGCAAAAAACGCCAACCTACAACCACCAGCCACAAACATGGAGCC
||

SNP:    IR-76
ACCESSION:
COMMENT:
        Forward PCR primer:GCCAAGAAGGTCTCAGGGAG
        Reverse PCR primer:GGCTCCATGTTTGTGGCTGG
        Chromosome- X
SAMPLESIZE:    8
LENGTH: ?
5'_FLANK:      GCCAAGAAGGTCTCAGGGAGCCCCCAGTCCCTCCTTGGCTGCCAGGAAAAGAGCGGGTCGGCCACCATGTTAAGA
OBSERVED:      G/C
3'_FLANK:
        AGGCGCAGGGATGTGCAGGCGGAAACCTCAAACCAATTCCGCTTGATGGCAGTAGAGCACCAAGAAAGGCAACTGCCAGGCTGCTGGGCGCTCTCCCTCTGCCC
TTCCAGAGGCAAAAAACGCCAACCTACAACCACCAGCCACAAACATGGAGCC
||

SNP:    IR-78
ACCESSION:
COMMENT:
        Forward PCR primer:GCCAAGAAGGTCTCAGGGAG
        Reverse PCR primer:GGCTCCATGTTTGTGGCTGG
        Chromosome- X
SAMPLESIZE:    8
LENGTH: ?
5'_FLANK:      GCCAAGAAGGTCTCAGGGAGCCCCCAGTCCCTCCTTGGCTGCCAGGAAAAGAGCGGGTCGGCCACCATGTTAAGAGA
OBSERVED:      G/A
3'_FLANK:
        GCGCAGGGATGTGCAGGCGGAAACCTCAAACCAATTCCGCTTGATGGCAGTAGAGCACCAAGAAAGGCAACTGCCAGGCTGCTGGGCGCTCTCCCTCTGCCCTT
CCAGAGGCAAAAAACGCCAACCTACAACCACCAGCCACAAACATGGAGCC
||

SNP:    IR-81
ACCESSION:
COMMENT:
        Forward PCR primer:GCCAAGAAGGTCTCAGGGAG
        Reverse PCR primer:GGCTCCATGTTTGTGGCTGG
        Chromosome- X
SAMPLESIZE:    8
LENGTH: ?
5'_FLANK:      GCCAAGAAGGTCTCAGGGAGCCCCCAGTCCCTCCTTGGCTGCCAGGAAAAGAGCGGGTCGGCCACCATGTTAAGAGAGGC
OBSERVED:      G/A/T
3'_FLANK:
        CAGGGATGTGCAGGCGGAAACCTCAAACCAATTCCGCTTGATGGCAGTAGAGCACCAAGAAAGGCAACTGCCAGGCTGCTGGGCGCTCTCCCTCTGCCCTTCCA
GAGGCAAAAAACGCCAACCTACAACCACCAGCCACAAACATGGAGCC
||

SNP:    IR-92
```

ACCESSION:
COMMENT:
Forward PCR primer:GCCAAGAAGGTCTCAGGGAG
Reverse PCR primer:GGCTCCATGTTTGTGGCTGG
Chromosome- X
SAMPLESIZE: 8
LENGTH: ?
5'_FLANK: GCCAAGAAGGTCTCAGGGAGCCCCCAGTCCCTCCTTGGCTGCCAGGAAAAGAGCGGGTCGGCCACCATGTTAAGAGAGGCGCAGGGATGTG
OBSERVED: C/A/T
3'_FLANK:
AGGCGGAAACCTCAAACCAATTCCGCTTGATGGCAGTAGAGCACCAAGAAAGGCAACTGCCAGGCTGCTGGGCGCTCTCCCTCTGCCCTTCCAGAGGCAAAAAA
CGCCAACCTACAACCACCAGCCACAAACATGGAGCC
||

SNP: IR-95
ACCESSION:
COMMENT:
Forward PCR primer:GCCAAGAAGGTCTCAGGGAG
Reverse PCR primer:GGCTCCATGTTTGTGGCTGG
Chromosome- X
SAMPLESIZE: 8
LENGTH: ?
5'_FLANK: GCCAAGAAGGTCTCAGGGAGCCCCCAGTCCCTCCTTGGCTGCCAGGAAAAGAGCGGGTCGGCCACCATGTTAAGAGAGGCGCAGGGATGTGCAG
OBSERVED: G/A
3'_FLANK:
CGGAAACCTCAAACCAATTCCGCTTGATGGCAGTAGAGCACCAAGAAAGGCAACTGCCAGGCTGCTGGGCGCTCTCCCTCTGCCCTTCCAGAGGCAAAAAACGC
CAACCTACAACCACCAGCCACAAACATGGAGCC
||

SNP: IR-97
ACCESSION:
COMMENT:
Forward PCR primer:GCCAAGAAGGTCTCAGGGAG
Reverse PCR primer:GGCTCCATGTTTGTGGCTGG
Chromosome- X
SAMPLESIZE: 8
LENGTH: ?
5'_FLANK: CCAAGAAGGTCTCAGGGAGCCCCCAGTCCCTCCTTGGCTGCCAGGAAAAGAGCGGGTCGGCCACCATGTTAAGAGAGGCGCAGGGATGTGCAGGC
OBSERVED: G/C
3'_FLANK:
GAAACCTCAAACCAATTCCGCTTGATGGCAGTAGAGCACCAAGAAAGGCAACTGCCAGGCTGCTGGGCGCTCTCCCTCTGCCCTTCCAGAGGCAAAAAACGCCA
ACCTACAACCACCAGCCACAAACATGGAGCC
||

SNP: IR-108
ACCESSION:
COMMENT:
Forward PCR primer:GCCAAGAAGGTCTCAGGGAG
Reverse PCR primer:GGCTCCATGTTTGTGGCTGG
Chromosome- X
SAMPLESIZE: 8
LENGTH: ?
5'_FLANK:
GCCAAGAAGGTCTCAGGGAGCCCCCAGTCCCTCCTTGGCTGCCAGGAAAAGAGCGGGTCGGCCACCATGTTAAGAGAGGCGCAGGGATGTGCAGGCGGAAACCT
CAA
OBSERVED: A/G
3'_FLANK:
CCAATTCCGCTTGATGGCAGTAGAGCACCAAGAAAGGCAACTGCCAGGCTGCTGGGCGCTCTCCCTCTGCCCTTCCAGAGGCAAAAAACGCCAACCTACAACCA
CCAGCCACAAACATGGAGCC
||

SNP: IR-111
ACCESSION:
COMMENT:
Forward PCR primer:GCCAAGAAGGTCTCAGGGAG
Reverse PCR primer:GGCTCCATGTTTGTGGCTGG
Chromosome- X
SAMPLESIZE: 8
LENGTH: ?
5'_FLANK:
GCCAAGAAGGTCTCAGGGAGCCCCCAGTCCCTCCTTGGCTGCCAGGAAAAGAGCGGGTCGGCCACCATGTTAAGAGAGGCGCAGGGATGTGCAGGCGGAAACCT
CAAACC
OBSERVED: A/T
3'_FLANK:
ATTCCGCTTGATGGCAGTAGAGCACCAAGAAAGGCAACTGCCAGGCTGCTGGGCGCTCTCCCTCTGCCCTTCCAGAGGCAAAAAACGCCAACCTACAACCACCA
GCCACAAACATGGAGCC
||

SNP: IR-112
ACCESSION:
COMMENT:

Forward PCR primer:GCCAAGAAGGTCTCAGGGAG
Reverse PCR primer:GGCTCCATGTTTGTGGCTGG
Chromosome- X
SAMPLESIZE: 8
LENGTH: ?
5'_FLANK:
GCCAAGAAGGTCTCAGGGAGCCCCCAGTCCCTCCTTGGCTGCCAGGAAAAGAGCGGGTCGGCCACCATGTTAAGAGAGGCGCAGGGATGTGCAGGCGGAAACCT
CAAACCA
OBSERVED: A/T
3'_FLANK:
TTCCGCTTGATGGCAGTAGAGCACCAAGAAAGGCAACTGCCAGGCTGCTGGGCGCTCTCCCTCTGCCCTTCCAGAGGCAAAAAACGCCAACCTACAACCACCAG
CCACAAACATGGAGCC
||

SNP: IR-114
ACCESSION:
COMMENT:
Forward PCR primer:GCCAAGAAGGTCTCAGGGAG
Reverse PCR primer:GGCTCCATGTTTGTGGCTGG
Chromosome- X
SAMPLESIZE: 8
LENGTH: ?
5'_FLANK:
GCCAAGAAGGTCTCAGGGAGCCCCCAGTCCCTCCTTGGCTGCCAGGAAAAGAGCGGGTCGGCCACCATGTTAAGAGAGGCGCAGGGATGTGCAGGCGGAAACCT
CAAACCAAT
OBSERVED: T/C
3'_FLANK:
CCGCTTGATGGCAGTAGAGCACCAAGAAAGGCAACTGCCAGGCTGCTGGGCGCTCTCCCTCTGCCCTTCCAGAGGCAAAAAACGCCAACCTACAACCACCAGCC
ACAAACATGGAGCC
||

SNP: IR-116
ACCESSION:
COMMENT:
Forward PCR primer:GCCAAGAAGGTCTCAGGGAG
Reverse PCR primer:GGCTCCATGTTTGTGGCTGG
Chromosome- X
SAMPLESIZE: 8
LENGTH: ?
5'_FLANK:
GCCAAGAAGGTCTCAGGGAGCCCCCAGTCCCTCCTTGGCTGCCAGGAAAAGAGCGGGTCGGCCACCATGTTAAGAGAGGCGCAGGGATGTGCAGGCGGAAACCT
CAAACCAATTC
OBSERVED: C/T
3'_FLANK:
GCTTGATGGCAGTAGAGCACCAAGAAAGGCAACTGCCAGGCTGCTGGGCGCTCTCCCTCTGCCCTTCCAGAGGCAAAAAACGCCAACCTACAACCACCAGCCAC
AAACATGGAGCC
||

SNP: IR-119
ACCESSION:
COMMENT:
Forward PCR primer:GCCAAGAAGGTCTCAGGGAG
Reverse PCR primer:GGCTCCATGTTTGTGGCTGG
Chromosome- X
SAMPLESIZE: 8
LENGTH: ?
5'_FLANK:
GCCAAGAAGGTCTCAGGGAGCCCCCAGTCCCTCCTTGGCTGCCAGGAAAAGAGCGGGTCGGCCACCATGTTAAGAGAGGCGCAGGGATGTGCAGGCGGAAACCT
CAAACCAATTCCGC
OBSERVED: T/G/C
3'_FLANK:
TGATGGCAGTAGAGCACCAAGAAAGGCAACTGCCAGGCTGCTGGGCGCTCTCCCTCTGCCCTTCCAGAGGCAAAAAACGCCAACCTACAACCACCAGCCACAAA
CATGGAGCC
||

SNP: IR-131
ACCESSION:
COMMENT:
Forward PCR primer:GCCAAGAAGGTCTCAGGGAG
Reverse PCR primer:GGCTCCATGTTTGTGGCTGG
Chromosome- X
SAMPLESIZE: 8
LENGTH: ?
5'_FLANK:
GCCAAGAAGGTCTCAGGGAGCCCCCAGTCCCTCCTTGGCTGCCAGGAAAAGAGCGGGTCGGCCACCATGTTAAGAGAGGCGCAGGGATGTGCAGGCGGAAACCT
CAAACCAATTCCGCTTGATGGCAGTA
OBSERVED: G/A
3'_FLANK:
AGCACCAAGAAAGGCAACTGCCAGGCTGCTGGGCGCTCTCCCTCTGCCCTTCCAGAGGCAAAAAACGCCAACCTACAACCACCAGCCACAAACATGGAGCC
||

SNP: IR-133
ACCESSION:
COMMENT:
Forward PCR primer:GCCAAGAAGGTCTCAGGGAG
Reverse PCR primer:GGCTCCATGTTTGTGGCTGG
Chromosome- X
SAMPLESIZE: 8
LENGTH: ?
5'_FLANK:
GCCAAGAAGGTCTCAGGGAGCCCCCAGTCCCTCCTTGGCTGCCAGGAAAAGAGCGGGTCGGCCACCATGTTAAGAGAGGCGCAGGGATGTGCAGGCGGAAACCT
CAAACCAATTCCGCTTGATGGCAGTAGA
OBSERVED: G/A
3'_FLANK:
CACCAAGAAAGGCAACTGCCAGGCTGCTGGGCGCTCTCCCTCTGCCCTTCCAGAGGCAAAAAACGCCAACCTACAACCACCAGCCACAAACATGGAGCC
||

SNP: IR-138
ACCESSION:
COMMENT:
Forward PCR primer:GCCAAGAAGGTCTCAGGGAG
Reverse PCR primer:GGCTCCATGTTTGTGGCTGG
Chromosome- X
SAMPLESIZE: 8
LENGTH: ?
5'_FLANK:
GCCAAGAAGGTCTCAGGGAGCCCCCAGTCCCTCCTTGGCTGCCAGGAAAAGAGCGGGTCGGCCACCATGTTAAGAGAGGCGCAGGGATGTGCAGGCGGAAACCT
CAAACCAATTCCGCTTGATGGCAGTAGAGCACC
OBSERVED: A/G
3'_FLANK: AGAAAGGCAACTGCCAGGCTGCTGGGCGCTCTCCCTCTGCCCTTCCAGAGGCAAAAAACGCCAACCTACAACCACCAGCCACAAACATGGAGCC
||

SNP: IR-139
ACCESSION:
COMMENT:
Forward PCR primer:GCCAAGAAGGTCTCAGGGAG
Reverse PCR primer:GGCTCCATGTTTGTGGCTGG
Chromosome- X
SAMPLESIZE: 8
LENGTH: ?
5'_FLANK:
GCCAAGAAGGTCTCAGGGAGCCCCCAGTCCCTCCTTGGCTGCCAGGAAAAGAGCGGGTCGGCCACCATGTTAAGAGAGGCGCAGGGATGTGCAGGCGGAAACCT
CAAACCAATTCCGCTTGATGGCAGTAGAGCACCA
OBSERVED: A/C/G
3'_FLANK: GAAAGGCAACTGCCAGGCTGCTGGGCGCTCTCCCTCTGCCCTTCCAGAGGCAAAAAACGCCAACCTACAACCACCAGCCACAAACATGGAGCC
||

SNP: IR-140
ACCESSION:
COMMENT:
Forward PCR primer:GCCAAGAAGGTCTCAGGGAG
Reverse PCR primer:GGCTCCATGTTTGTGGCTGG
Chromosome- X
SAMPLESIZE: 8
LENGTH: ?
5'_FLANK:
GCCAAGAAGGTCTCAGGGAGCCCCCAGTCCCTCCTTGGCTGCCAGGAAAAGAGCGGGTCGGCCACCATGTTAAGAGAGGCGCAGGGATGTGCAGGCGGAAACCT
CAAACCAATTCCGCTTGATGGCAGTAGAGCACCAA
OBSERVED: G/A
3'_FLANK: AAAGGCAACTGCCAGGCTGCTGGGCGCTCTCCCTCTGCCCTTCCAGAGGCAAAAAACGCCAACCTACAACCACCAGCCACAAACATGGAGCC
||

SNP: IR-141
ACCESSION:
COMMENT:
Forward PCR primer:GCCAAGAAGGTCTCAGGGAG
Reverse PCR primer:GGCTCCATGTTTGTGGCTGG
Chromosome- X
SAMPLESIZE: 8
LENGTH: ?
5'_FLANK:
GCCAAGAAGGTCTCAGGGAGCCCCCAGTCCCTCCTTGGCTGCCAGGAAAAGAGCGGGTCGGCCACCATGTTAAGAGAGGCGCAGGGATGTGCAGGCGGAAACCT
CAAACCAATTCCGCTTGATGGCAGTAGAGCACCAAG
OBSERVED: A/G
3'_FLANK: AAGGCAACTGCCAGGCTGCTGGGCGCTCTCCCTCTGCCCTTCCAGAGGCAAAAAACGCCAACCTACAACCACCAGCCACAAACATGGAGCC
||

SNP: IR-143
ACCESSION:
COMMENT:
Forward PCR primer:GCCAAGAAGGTCTCAGGGAG
Reverse PCR primer:GGCTCCATGTTTGTGGCTGG

Chromosome- X
SAMPLESIZE: 8
LENGTH: ?
5'_FLANK:
GCCAAGAAGGTCTCAGGGAGCCCCCAGTCCCTCCTTGGCTGCCAGGAAAAGAGCGGGTCGGCCACCATGTTAAGAGAGGCGCAGGGATGTGCAGGCGGAAACCT
CAAACCAATTCCGCTTGATGGCAGTAGAGCACCAAGAA
OBSERVED: A/T
3'_FLANK: GGCAACTGCCAGGCTGCTGGGCGCTCTCCCTCTGCCCTTCCAGAGGCAAAAAACGCCAACCTACAACCACCAGCCACAAACATGGAGCC
||
#
#SNP:
#ACCESSION:
#COMMENT:
Forward PCR primer:GCCAAGAAGGTCTCAGGGAG
Reverse PCR primer:GGCTCCATGTTTGTGGCTGG
Chromosome- X
#SAMPLESIZE: 8
#LENGTH: ?
#5'_FLANK:
#OBSERVED:
#3'_FLANK:
#||

SNP: IR-147
ACCESSION:
COMMENT:
Forward PCR primer:GCCAAGAAGGTCTCAGGGAG
Reverse PCR primer:GGCTCCATGTTTGTGGCTGG
Chromosome- X
SAMPLESIZE: 8
LENGTH: ?
5'_FLANK:
GCCAAGAAGGTCTCAGGGAGCCCCCAGTCCCTCCTTGGCTGCCAGGAAAAGAGCGGGTCGGCCACCATGTTAAGAGAGGCGCAGGGATGTGCAGGCGGAAACCT
CAAACCAATTCCGCTTGATGGCAGTAGAGCACCAAGAAAGGC
OBSERVED: A/G
3'_FLANK: ACTGCCAGGCTGCTGGGCGCTCTCCCTCTGCCCTTCCAGAGGCAAAAAACGCCAACCTACAACCACCAGCCACAAACATGGAGCC
||

SNP: IR-150
ACCESSION:
COMMENT:
Forward PCR primer:GCCAAGAAGGTCTCAGGGAG
Reverse PCR primer:GGCTCCATGTTTGTGGCTGG
Chromosome- X
SAMPLESIZE: 8
LENGTH: ?
5'_FLANK:
GCCAAGAAGGTCTCAGGGAGCCCCCAGTCCCTCCTTGGCTGCCAGGAAAAGAGCGGGTCGGCCACCATGTTAAGAGAGGCGCAGGGATGTGCAGGCGGAAACCT
CAAACCAATTCCGCTTGATGGCAGTAGAGCACCAAGAAAGGCAAC
OBSERVED: T/C
3'_FLANK: GCCAGGCTGCTGGGCGCTCTCCCTCTGCCCTTCCAGAGGCAAAAAACGCCAACCTACAACCACCAGCCACAAACATGGAGCC
||

SNP: IR-153
ACCESSION:
COMMENT:
Forward PCR primer:GCCAAGAAGGTCTCAGGGAG
Reverse PCR primer:GGCTCCATGTTTGTGGCTGG
Chromosome- X
SAMPLESIZE: 8
LENGTH: ?
5'_FLANK:
GCCAAGAAGGTCTCAGGGAGCCCCCAGTCCCTCCTTGGCTGCCAGGAAAAGAGCGGGTCGGCCACCATGTTAAGAGAGGCGCAGGGATGTGCAGGCGGAAACCT
CAAACCAATTCCGCTTGATGGCAGTAGAGCACCAAGAAAGGCAACTGC
OBSERVED: C/T
3'_FLANK: AGGCTGCTGGGCGCTCTCCCTCTGCCCTTCCAGAGGCAAAAAACGCCAACCTACAACCACCAGCCACAAACATGGAGCC
||

SNP: IR-155
ACCESSION:
COMMENT:
Forward PCR primer:GCCAAGAAGGTCTCAGGGAG
Reverse PCR primer:GGCTCCATGTTTGTGGCTGG
Chromosome- X
SAMPLESIZE: 8
LENGTH: ?
5'_FLANK:
GCCAAGAAGGTCTCAGGGAGCCCCCAGTCCCTCCTTGGCTGCCAGGAAAAGAGCGGGTCGGCCACCATGTTAAGAGAGGCGCAGGGATGTGCAGGCGGAAACCT
CAAACCAATTCCGCTTGATGGCAGTAGAGCACCAAGAAAGGCAACTGCCA
OBSERVED: G/C/A
3'_FLANK: GCTGCTGGGCGCTCTCCCTCTGCCCTTCCAGAGGCAAAAAACGCCAACCTACAACCACCAGCCACAAACATGGAGCC

||

SNP: IR-158
ACCESSION:
COMMENT:
Forward PCR primer:GCCAAGAAGGTCTCAGGGAG
Reverse PCR primer:GGCTCCATGTTTGTGGCTGG
Chromosome- X
SAMPLESIZE: 8
LENGTH: ?
5'_FLANK:
GCCAAGAAGGTCTCAGGGAGCCCCCAGTCCCTCCTTGGCTGCCAGGAAAAGAGCGGGTCGGCCACCATGTTAAGAGAGGCGCAGGGATGTGCAGGCGGAAACCT
CAAACCAATTCCGCTTGATGGCAGTAGAGCACCAAGAAAGGCAACTGCCAGGC
OBSERVED: T/G
3'_FLANK: GCTGGGCGCTCTCCCTCTGCCCTTCCAGAGGCAAAAAACGCCAACCTACAACCACCAGCCACAAACATGGAGCC
||

SNP: IR-165
ACCESSION:
COMMENT:
Forward PCR primer:GCCAAGAAGGTCTCAGGGAG
Reverse PCR primer:GGCTCCATGTTTGTGGCTGG
Chromosome- X
SAMPLESIZE: 8
LENGTH: ?
5'_FLANK:
GCCAAGAAGGTCTCAGGGAGCCCCCAGTCCCTCCTTGGCTGCCAGGAAAAGAGCGGGTCGGCCACCATGTTAAGAGAGGCGCAGGGATGTGCAGGCGGAAACCT
CAAACCAATTCCGCTTGATGGCAGTAGAGCACCAAGAAAGGCAACTGCCAGGCTGCTGGG
OBSERVED: C/T
3'_FLANK: GCTCTCCCTCTGCCCTTCCAGAGGCAAAAAACGCCAACCTACAACCACCAGCCACAAACATGGAGCC
||

SNP: IR-166
ACCESSION:
COMMENT:
Forward PCR primer:GCCAAGAAGGTCTCAGGGAG
Reverse PCR primer:GGCTCCATGTTTGTGGCTGG
Chromosome- X
SAMPLESIZE: 8
LENGTH: ?
5'_FLANK:
GCCAAGAAGGTCTCAGGGAGCCCCCAGTCCCTCCTTGGCTGCCAGGAAAAGAGCGGGTCGGCCACCATGTTAAGAGAGGCGCAGGGATGTGCAGGCGGAAACCT
CAAACCAATTCCGCTTGATGGCAGTAGAGCACCAAGAAAGGCAACTGCCAGGCTGCTGGGC
OBSERVED: G/A
3'_FLANK: CTCTCCCTCTGCCCTTCCAGAGGCAAAAAACGCCAACCTACAACCACCAGCCACAAACATGGAGCC
||

SNP: IR-167
ACCESSION:
COMMENT:
Forward PCR primer:GCCAAGAAGGTCTCAGGGAG
Reverse PCR primer:GGCTCCATGTTTGTGGCTGG
Chromosome- X
SAMPLESIZE: 8
LENGTH: ?
5'_FLANK:
GCCAAGAAGGTCTCAGGGAGCCCCCAGTCCCTCCTTGGCTGCCAGGAAAAGAGCGGGTCGGCCACCATGTTAAGAGAGGCGCAGGGATGTGCAGGCGGAAACCT
CAAACCAATTCCGCTTGATGGCAGTAGAGCACCAAGAAAGGCAACTGCCAGGCTGCTGGGCG
OBSERVED: C/T/A
3'_FLANK: TCTCCCTCTGCCCTTCCAGAGGCAAAAAACGCCAACCTACAACCACCAGCCACAAACATGGAGCC
||

SNP: IR-168
ACCESSION:
COMMENT:
Forward PCR primer:GCCAAGAAGGTCTCAGGGAG
Reverse PCR primer:GGCTCCATGTTTGTGGCTGG
Chromosome- X
SAMPLESIZE: 8
LENGTH: ?
5'_FLANK:
GCCAAGAAGGTCTCAGGGAGCCCCCAGTCCCTCCTTGGCTGCCAGGAAAAGAGCGGGTCGGCCACCATGTTAAGAGAGGCGCAGGGATGTGCAGGCGGAAACCT
CAAACCAATTCCGCTTGATGGCAGTAGAGCACCAAGAAAGGCAACTGCCAGGCTGCTGGGCGC
OBSERVED: T/G
3'_FLANK: CTCCCTCTGCCCTTCCAGAGGCAAAAAACGCCAACCTACAACCACCAGCCACAAACATGGAGCC
||

SNP: IR-169
ACCESSION:
COMMENT:
Forward PCR primer:GCCAAGAAGGTCTCAGGGAG

Reverse PCR primer:GGCTCCATGTTTGTGGCTGG
Chromosome- X
SAMPLESIZE: 8
LENGTH: ?
5'_FLANK:
GCCAAGAAGGTCTCAGGGAGCCCCCAGTCCCTCCTTGGCTGCCAGGAAAAGAGCGGGTCGGCCACCATGTTAAGAGAGGCGCAGGGATGTGCAGGCGGAAACCT
CAAACCAATTCCGCTTGATGGCAGTAGAGCACCAAGAAAGGCAACTGCCAGGCTGCTGGGCGCT
OBSERVED: C/T
3'_FLANK: TCCCTCTGCCCTTCCAGAGGCAAAAAACGCCAACCTACAACCACCAGCCACAAACATGGAGCC
||

SNP: IR-174
ACCESSION:
COMMENT:
Forward PCR primer:GCCAAGAAGGTCTCAGGGAG
Reverse PCR primer:GGCTCCATGTTTGTGGCTGG
Chromosome- X
SAMPLESIZE: 8
LENGTH: ?
5'_FLANK:
GCCAAGAAGGTCTCAGGGAGCCCCCAGTCCCTCCTTGGCTGCCAGGAAAAGAGCGGGTCGGCCACCATGTTAAGAGAGGCGCAGGGATGTGCAGGCGGAAACCT
CAAACCAATTCCGCTTGATGGCAGTAGAGCACCAAGAAAGGCAACTGCCAGGCTGCTGGGCGCTCTCCC
OBSERVED: T/C
3'_FLANK: CTGCCCTTCCAGAGGCAAAAAACGCCAACCTACAACCACCAGCCACAAACATGGAGCC
||

SNP: IR-176
ACCESSION:
COMMENT:
Forward PCR primer:GCCAAGAAGGTCTCAGGGAG
Reverse PCR primer:GGCTCCATGTTTGTGGCTGG
Chromosome- X
SAMPLESIZE: 8
LENGTH: ?
5'_FLANK:
GCCAAGAAGGTCTCAGGGAGCCCCCAGTCCCTCCTTGGCTGCCAGGAAAAGAGCGGGTCGGCCACCATGTTAAGAGAGGCGCAGGGATGTGCAGGCGGAAACCT
CAAACCAATTCCGCTTGATGGCAGTAGAGCACCAAGAAAGGCAACTGCCAGGCTGCTGGGCGCTCTCCCTC
OBSERVED: T/C
3'_FLANK: GCCCTTCCAGAGGCAAAAAACGCCAACCTACAACCACCAGCCACAAACATGGAGCC
||

SNP: IR-177
ACCESSION:
COMMENT:
Forward PCR primer:GCCAAGAAGGTCTCAGGGAG
Reverse PCR primer:GGCTCCATGTTTGTGGCTGG
Chromosome- X
SAMPLESIZE: 8
LENGTH: ?
5'_FLANK:
GCCAAGAAGGTCTCAGGGAGCCCCCAGTCCCTCCTTGGCTGCCAGGAAAAGAGCGGGTCGGCCACCATGTTAAGAGAGGCGCAGGGATGTGCAGGCGGAAACCT
CAAACCAATTCCGCTTGATGGCAGTAGAGCACCAAGAAAGGCAACTGCCAGGCTGCTGGGCGCTCTCCCTCT
OBSERVED: G/T
3'_FLANK: CCCTTCCAGAGGCAAAAAACGCCAACCTACAACCACCAGCCACAAACATGGAGCC
||

SNP: IR-179
ACCESSION:
COMMENT:
Forward PCR primer:GCCAAGAAGGTCTCAGGGAG
Reverse PCR primer:GGCTCCATGTTTGTGGCTGG
Chromosome- X
SAMPLESIZE: 8
LENGTH: ?
5'_FLANK:
GCCAAGAAGGTCTCAGGGAGCCCCCAGTCCCTCCTTGGCTGCCAGGAAAAGAGCGGGTCGGCCACCATGTTAAGAGAGGCGCAGGGATGTGCAGGCGGAAACCT
CAAACCAATTCCGCTTGATGGCAGTAGAGCACCAAGAAAGGCAACTGCCAGGCTGCTGGGCGCTCTCCCTCTGC
OBSERVED: C/T
3'_FLANK: CTTCCAGAGGCAAAAAACGCCAACCTACAACCACCAGCCACAAACATGGAGCC
||

SNP: IR-181
ACCESSION:
COMMENT:
Forward PCR primer:GCCAAGAAGGTCTCAGGGAG
Reverse PCR primer:GGCTCCATGTTTGTGGCTGG
Chromosome- X
SAMPLESIZE: 8
LENGTH: ?

```
5'_FLANK:
        GCCAAGAAGGTCTCAGGGAGCCCCCAGTCCCTCCTTGGCTGCCAGGAAAAGAGCGGGTCGGCCACCATGTTAAGAGAGGCGCAGGGATGTGCAGGCGGAAACCT
CAAACCAATTCCGCTTGATGGCAGTAGAGCACCAAGAAAGGCAACTGCCAGGCTGCTGGGCGCTCTCCCTCTGCCC
OBSERVED:       T/G
3'_FLANK:       TCCAGAGGCAAAAAACGCCAACCTACAACCACCAGCCACAAACATGGAGCC
||

SNP:    IR-182
ACCESSION:
COMMENT:
        Forward PCR primer:GCCAAGAAGGTCTCAGGGAG
        Reverse PCR primer:GGCTCCATGTTTGTGGCTGG
        Chromosome- X
SAMPLESIZE:     8
LENGTH: ?
5'_FLANK:
        GCCAAGAAGGTCTCAGGGAGCCCCCAGTCCCTCCTTGGCTGCCAGGAAAAGAGCGGGTCGGCCACCATGTTAAGAGAGGCGCAGGGATGTGCAGGCGGAAACCT
CAAACCAATTCCGCTTGATGGCAGTAGAGCACCAAGAAAGGCAACTGCCAGGCTGCTGGGCGCTCTCCCTCTGCCCT
OBSERVED:       T/C
3'_FLANK:       CCAGAGGCAAAAAACGCCAACCTACAACCACCAGCCACAAACATGGAGCC
||

SNP:    IR-183
ACCESSION:
COMMENT:
        Forward PCR primer:GCCAAGAAGGTCTCAGGGAG
        Reverse PCR primer:GGCTCCATGTTTGTGGCTGG
        Chromosome- X
SAMPLESIZE:     8
LENGTH: ?
5'_FLANK:
        GCCAAGAAGGTCTCAGGGAGCCCCCAGTCCCTCCTTGGCTGCCAGGAAAAGAGCGGGTCGGCCACCATGTTAAGAGAGGCGCAGGGATGTGCAGGCGGAAACCT
CAAACCAATTCCGCTTGATGGCAGTAGAGCACCAAGAAAGGCAACTGCCAGGCTGCTGGGCGCTCTCCCTCTGCCCTT
OBSERVED:       C/T
3'_FLANK:       CAGAGGCAAAAAACGCCAACCTACAACCACCAGCCACAAACATGGAGCC
||

SNP:    IR-184
ACCESSION:
COMMENT:
        Forward PCR primer:GCCAAGAAGGTCTCAGGGAG
        Reverse PCR primer:GGCTCCATGTTTGTGGCTGG
        Chromosome- X
SAMPLESIZE:     8
LENGTH: ?
5'_FLANK:
        GCCAAGAAGGTCTCAGGGAGCCCCCAGTCCCTCCTTGGCTGCCAGGAAAAGAGCGGGTCGGCCACCATGTTAAGAGAGGCGCAGGGATGTGCAGGCGGAAACCT
CAAACCAATTCCGCTTGATGGCAGTAGAGCACCAAGAAAGGCAACTGCCAGGCTGCTGGGCGCTCTCCCTCTGCCCTTC
OBSERVED:       C/T
3'_FLANK:       AGAGGCAAAAAACGCCAACCTACAACCACCAGCCACAAACATGGAGCC
||

SNP:    IR-187
ACCESSION:
COMMENT:
        Forward PCR primer:GCCAAGAAGGTCTCAGGGAG
        Reverse PCR primer:GGCTCCATGTTTGTGGCTGG
        Chromosome- X
SAMPLESIZE:     8
LENGTH: ?
5'_FLANK:
        GCCAAGAAGGTCTCAGGGAGCCCCCAGTCCCTCCTTGGCTGCCAGGAAAAGAGCGGGTCGGCCACCATGTTAAGAGAGGCGCAGGGATGTGCAGGCGGAAACCT
CAAACCAATTCCGCTTGATGGCAGTAGAGCACCAAGAAAGGCAACTGCCAGGCTGCTGGGCGCTCTCCCTCTGCCCTTCCAG
OBSERVED:       A/T
3'_FLANK:       GGCAAAAAACGCCAACCTACAACCACCAGCCACAAACATGGAGCC
||

SNP:    IR-195
ACCESSION:
COMMENT:
        Forward PCR primer:GCCAAGAAGGTCTCAGGGAG
        Reverse PCR primer:GGCTCCATGTTTGTGGCTGG
        Chromosome- X
SAMPLESIZE:     8
LENGTH: ?
5'_FLANK:
        GCCAAGAAGGTCTCAGGGAGCCCCCAGTCCCTCCTTGGCTGCCAGGAAAAGAGCGGGTCGGCCACCATGTTAAGAGAGGCGCAGGGATGTGCAGGCGGAAACCT
CAAACCAATTCCGCTTGATGGCAGTAGAGCACCAAGAAAGGCAACTGCCAGGCTGCTGGGCGCTCTCCCTCTGCCCTTCCAGAGGCAAAA
OBSERVED:       A/G
3'_FLANK:       ACGCCAACCTACAACCACCAGCCACAAACATGGAGCC
||
```

SNP: IR-197
ACCESSION:
COMMENT:
Forward PCR primer:GCCAAGAAGGTCTCAGGGAG
Reverse PCR primer:GGCTCCATGTTTGTGGCTGG
Chromosome- X
SAMPLESIZE: 8
LENGTH: ?
5'_FLANK:
GCCAAGAAGGTCTCAGGGAGCCCCCAGTCCCTCCTTGGCTGCCAGGAAAAGAGCGGGTCGGCCACCATGTTAAGAGAGGCGCAGGGATGTGCAGGCGGAAACCT
CAAACCAATTCCGCTTGATGGCAGTAGAGCACCAAGAAAGGCAACTGCCAGGCTGCTGGGCGCTCTCCCTCTGCCCTTCCAGAGGCAAAAAA
OBSERVED: C/T
3'_FLANK: GCCAACCTACAACCACCAGCCACAAACATGGAGCC
||

SNP: IR-201
ACCESSION:
COMMENT:
Forward PCR primer:GCCAAGAAGGTCTCAGGGAG
Reverse PCR primer:GGCTCCATGTTTGTGGCTGG
Chromosome- X
SAMPLESIZE: 8
LENGTH: ?
5'_FLANK:
GCCAAGAAGGTCTCAGGGAGCCCCCAGTCCCTCCTTGGCTGCCAGGAAAAGAGCGGGTCGGCCACCATGTTAAGAGAGGCGCAGGGATGTGCAGGCGGAAACCT
CAAACCAATTCCGCTTGATGGCAGTAGAGCACCAAGAAAGGCAACTGCCAGGCTGCTGGGCGCTCTCCCTCTGCCCTTCCAGAGGCAAAAAACGCC
OBSERVED: A/G
3'_FLANK: ACCTACAACCACCAGCCACAAACATGGAGCC
||

SNP: IR-204
ACCESSION:
COMMENT:
Forward PCR primer:GCCAAGAAGGTCTCAGGGAG
Reverse PCR primer:GGCTCCATGTTTGTGGCTGG
Chromosome- X
SAMPLESIZE: 8
LENGTH: ?
5'_FLANK:
GCCAAGAAGGTCTCAGGGAGCCCCCAGTCCCTCCTTGGCTGCCAGGAAAAGAGCGGGTCGGCCACCATGTTAAGAGAGGCGCAGGGATGTGCAGGCGGAAACCT
CAAACCAATTCCGCTTGATGGCAGTAGAGCACCAAGAAAGGCAACTGCCAGGCTGCTGGGCGCTCTCCCTCTGCCCTTCCAGAGGCAAAAAACGCCAAC
OBSERVED: C/A
3'_FLANK: TACAACCACCAGCCACAAACATGGAGCC
||

SNP: IR-206
ACCESSION:
COMMENT:
Forward PCR primer:GCCAAGAAGGTCTCAGGGAG
Reverse PCR primer:GGCTCCATGTTTGTGGCTGG
Chromosome- X
SAMPLESIZE: 8
LENGTH: ?
5'_FLANK:
GCCAAGAAGGTCTCAGGGAGCCCCCAGTCCCTCCTTGGCTGCCAGGAAAAGAGCGGGTCGGCCACCATGTTAAGAGAGGCGCAGGGATGTGCAGGCGGAAACCT
CAAACCAATTCCGCTTGATGGCAGTAGAGCACCAAGAAAGGCAACTGCCAGGCTGCTGGGCGCTCTCCCTCTGCCCTTCCAGAGGCAAAAAACGCCAACCT
OBSERVED: A/G
3'_FLANK: CAACCACCAGCCACAAACATGGAGCC
||

SNP: IR-209
ACCESSION:
COMMENT:
Forward PCR primer:GCCAAGAAGGTCTCAGGGAG
Reverse PCR primer:GGCTCCATGTTTGTGGCTGG
Chromosome- X
SAMPLESIZE: 8
LENGTH: ?
5'_FLANK:
GCCAAGAAGGTCTCAGGGAGCCCCCAGTCCCTCCTTGGCTGCCAGGAAAAGAGCGGGTCGGCCACCATGTTAAGAGAGGCGCAGGGATGTGCAGGCGGAAACCT
CAAACCAATTCCGCTTGATGGCAGTAGAGCACCAAGAAAGGCAACTGCCAGGCTGCTGGGCGCTCTCCCTCTGCCCTTCCAGAGGCAAAAAACGCCAACCTACA
OBSERVED: A/T
3'_FLANK: CCACCAGCCACAAACATGGAGCCTGAGCACTAGGCCTTCCACTGGGCAGAATGTCTCTCTGTGCCCTGC
||

SNP: IR-210
ACCESSION:
COMMENT:
Forward PCR primer:GCCAAGAAGGTCTCAGGGAG
Reverse PCR primer:GGCTCCATGTTTGTGGCTGG

Chromosome- X
SAMPLESIZE: 8
LENGTH: ?
5'_FLANK:
GCCAAGAAGGTCTCAGGGAGCCCCCAGTCCCTCCTTGGCTGCCAGGAAAAGAGCGGGTCGGCCACCATGTTAAGAGAGGCGCAGGGATGTGCAGGCGGAAACCT
CAAACCAATTCCGCTTGATGGCAGTAGAGCACCAAGAAAGGCAACTGCCAGGCTGCTGGGCGCTCTCCCTCTGCCCTTCCAGAGGCAAAAAACGCCAACCTACAA
OBSERVED: C/T
3'_FLANK: CACCAGCCACAAACATGGAGCCTGAGCACTAGGCCTTCCACTGGGCAGAATGTCTCTCTGTGCCCTGC
||

SNP: IR-211
ACCESSION:
COMMENT:
Forward PCR primer:GCCAAGAAGGTCTCAGGGAG
Reverse PCR primer:GGCTCCATGTTTGTGGCTGG
Chromosome- X
SAMPLESIZE: 8
LENGTH: ?
5'_FLANK:
GCCAAGAAGGTCTCAGGGAGCCCCCAGTCCCTCCTTGGCTGCCAGGAAAAGAGCGGGTCGGCCACCATGTTAAGAGAGGCGCAGGGATGTGCAGGCGGAAACCT
CAAACCAATTCCGCTTGATGGCAGTAGAGCACCAAGAAAGGCAACTGCCAGGCTGCTGGGCGCTCTCCCTCTGCCCTTCCAGAGGCAAAAAACGCCAACCTACAAC
OBSERVED: C/G
3'_FLANK: ACCAGCCACAAACATGGAGCCTGAGCACTAGGCCTTCCACTGGGCAGAATGTCTCTCTGTGCCCTGC

||
SNP: Indel-55
ACCESSION:
SAMPLESIZE: 8
LENGTH: ?
5'_FLANK: GCCAAGAAGGTCTCAGGGAGCCCCCAGTCCCTCCTTGGCTGCCAGGAAAAGAGC
OBSERVED: G/-
3'_FLANK: GGTCGGCCACCATGTTAAGAGAGGCGCAGGGATGTGCAGGCGGAAACCTCAAACCAATTCCGCTT
||
#||
SNP: Indel-190
ACCESSION:
SAMPLESIZE: 8
LENGTH: ?
5'_FLANK:
GCCAAGAAGGTCTCAGGGAGCCCCCAGTCCCTCCTTGGCTGCCAGGAAAAGAGCGGGTCGGCCACCATGTTAAGAGAGGCGCAGGGATGTGCAGGCGGAAACCT
CAAACCAATTCCGCTTGATGGCAGTAGAGCACCAAGAAAGGCAACTGCCAGGCTGCTGGGCGCTCTCCCTCTGCCCTTCCAGAGG
OBSERVED: C/-
3'_FLANK: AAAAAACGCCAACCTACAACCACCAGCCACAAACATGGAGCCTGAGCACTAGGCCTTCCACTGGGCAGAATGTCTCTCTGTGCCCTGC
#||
||
SNP: Indel-196
ACCESSION:
SAMPLESIZE: 8
LENGTH: ?
5'_FLANK:
GCCAAGAAGGTCTCAGGGAGCCCCCAGTCCCTCCTTGGCTGCCAGGAAAAGAGCGGGTCGGCCACCATGTTAAGAGAGGCGCAGGGATGTGCAGGCGGAAACCT
CAAACCAATTCCGCTTGATGGCAGTAGAGCACCAAGAAAGGCAACTGCCAGGCTGCTGGGCGCTCTCCCTCTGCCCTTCCAGAGGCAAAAAA
OBSERVED: A/AA/AAC/AAAC/-
3'_FLANK: CGCCAACCTACAACCACCAGCCACAAACATGGAGCCTGAGCACTAGGCCTTCCACTGGGCAGAATGTCTCTCTGTGCCCTGC
||
